# Efficient DNP at high fields and fast MAS with antenna-sensitized dinitroxides†

**DOI:** 10.1039/d4sc04473h

**Published:** 2024-09-12

**Authors:** Lorenzo Niccoli, Gilles Casano, Georges Menzildjian, Maxim Yulikov, Thomas Robinson, Salah-Eddine Akrial, Zhuoran Wang, Christian Reiter, Armin Purea, Didier Siri, Amrit Venkatesh, Lyndon Emsley, David Gajan, Moreno Lelli, Olivier Ouari, Anne Lesage

**Affiliations:** a Centre de RMN à Hauts Champs de Lyon, UMR 5082, Université de Lyon (CNRS/ENS Lyon/UCBL) 5 rue de la Doua Villeurbanne 69100 France anne.lesage@ens-lyon.fr; b Center of Magnetic Resonance (CERM), University of Florence 50019 Sesto Fiorentino Italy; c Department of Chemistry ‘Ugo Schiff’, University of Florence Via della Lastruccia 13 50019 Sesto Fiorentino FI Italy; d Consorzio Interuniversitario Risonanze Magnetiche Metalloproteine Paramagnetiche (CIRMMP) Via Luigi Sacconi 6 50019 Sesto Fiorentino FI Italy moreno.lelli@unifi.it; e Aix Marseille Uni, CNRS, ICR 13013 Marseille France olivier.ouari@univ-amu.fr; f Department of Chemistry and Applied Biosciences, Eidgenössische Technische Hochschule Zürich CH-8093 Zürich Switzerland; g Bruker Biospin 76275 Ettlingen Germany; h Laboratory of Magnetic Resonance, Institut des Sciences et Ingénierie Chimiques, École Polytechnique Fédérale de Lausanne (EPFL) CH-1015 Lausanne Switzerland; i National High Magnetic Field Laboratory, Florida State University Tallahassee FL 32310 USA

## Abstract

Dynamic Nuclear Polarization (DNP) can significantly enhance the sensitivity of solid-state NMR. In DNP, microwave irradiation induces polarization transfer from unpaired electron spins to ^1^H nuclear spins *via* hyperfine couplings and spin-diffusion. The structure of the polarizing agents that host the electron spins is key for DNP efficiency. Currently, only a handful of structures perform well at very high magnetic fields (≥18.8 T), and enhancements are significantly lower than those obtained at lower fields. Here, we introduce a new series of water-soluble nitroxide biradicals with a scaffold augmented by dihydroxypropyl antenna chains that perform significantly better than previous dinitroxides at 18.8 T. The new radical M-TinyPol(OH)_4_ yields enhancement factors of ∼220 at 18.8 T and 60 kHz MAS, which is a nearly factor 2 larger than for the previous best performing dinitroxides. The performance is understood through ^2^H ESEEM measurements to probe solvent accessibility, supported by Molecular Dynamics simulations, and by experiments on deuterated samples. We find that the deuterated glycerol molecules in the matrix are located mainly in the second solvation shell of the NO bond, limiting access for protonated water molecules, and restricting spin diffusion pathways. This provides a rational understanding of why the dihydroxypropyl chains present in the best-performing structures are essential to deliver the polarization to the bulk solution.

## Abstract 2

Optimized dinitroxide structures by design for DNP enhanced solid-state NMR at high magnetic field.

## Graphical abstract

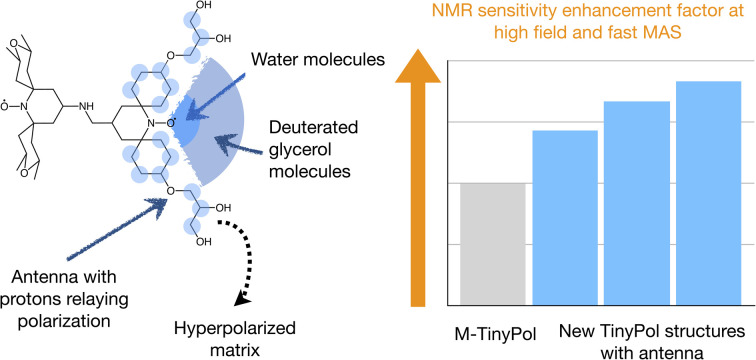

## Introduction

Solid-state magic angle spinning (MAS) nuclear magnetic resonance (NMR) spectroscopy is a powerful analytical technique that can provide atomic-level structural information on a broad range of solid substrates.^1^ Despite its high versatility, MAS NMR inherently lacks sensitivity due to low nuclear spin polarization at thermal equilibrium. To remediate this limitation significant developments have been made with microwave-driven dynamic nuclear polarization (DNP) in the last decades.^2–6^ DNP routinely yields ∼100-fold NMR signal amplification at cryogenic temperatures, and DNP enhanced solid-state NMR has already enabled many challenging new applications, overcoming the limitations of conventional MAS NMR spectroscopy, in research fields as diverse as nanomaterials, batteries, pharmaceuticals, polymers, supported catalysts, biomaterials, *in vitro* and *in cell* biomolecules.^7–25^ This boost in sensitivity is typically achieved by doping the substrate of interest with a polarizing agent (PA) containing unpaired electron spins, which transfer their significantly larger spin polarization to the surrounding nuclear spins under microwave irradiation.

Today, a large library of PAs with different molecular scaffolds have been introduced, with the objective of both increasing DNP efficiency and versatility.^26–54^ These range from hybrid molecular structures coupling a narrow electron paramagnetic resonance (EPR) line radical, such as tetrathiatriarylmethyl-based trityls or BDPA, with a nitroxide,^34,40,42,44,47,51^ to metal ion complexes,^41,43,49^ mixed-valence compounds,^37,48^ or biradicals based on two tethered nitroxides.^26–33,35,36,38,39,45,46,50,52,54^ These latter PAs rely on the cross-effect (CE) mechanism to transfer polarization.^55,56^ This transfer scheme is effective when the so-called CE matching condition is satisfied, *i.e.* when the difference between the Larmor frequencies of two unpaired electron spins matches the Larmor frequency of the target nucleus,^57–60^ which for dinitroxides is achieved as a result of their wide EPR line, that is inhomogeneously broadened by the anisotropy of the *g*-tensors. The efficiency of the CE mechanism depends on the strength of hyperfine coupling between the nucleus and one of the two electrons as well as dipolar and exchange couplings between the two electrons. Under MAS conditions, the magnetic field dependence of CE DNP becomes quite complex due to the interplay between adiabatic crossing events.^58^ The efficiency decreases following a trend between B_0_^−1^ and B_0_^−3^, depending on the specific parameters of the radical.^61^ Following the introduction of the first generation of dinitroxides,^26–28^ TEKPol^30^ and AMUPol^32^ were introduced based on key design concepts in the early 2010s. Under standard MAS DNP conditions at 9.4 T and sample temperatures of 100 K they yield more than 200-fold proton NMR signal enhancements (*ε*_H_) in frozen glassy solutions, (and up to a factor 350 with optimized instrumentation^62^). In the following years, attention has been dedicated to further improving the overall sensitivity gain^63–65^ in MAS DNP experiments through a detailed understanding of the parameters that drive PA efficiency. In 2016, Kubicki *et al.* compared a collection of more than 30 dinitroxides derived from a variety of molecular scaffolds, including bTurea, PyPol, and bTbk.^35^ In another systematic study, Sauvée *et al.* focused on 18 water-soluble bTurea derivatives, functionalized with a range of bulky groups around the N–O moiety or with various substituents on the linker so as to drive design principles.^33^ AsymPol radicals featuring a short tether and a conjugated carbon–carbon double bond in a five-membered ring nitroxide, were later introduced.^39^ These investigations, backed up with numerical studies^66–70^ established that sizeable electron–electron couplings, near-orthogonal *g*-tensors, as well as long electron relaxation times can constructively add up to improve the DNP performance of dinitroxides. Lately, the local geometry around the unpaired electrons has been identified as an additional parameter that governs the efficiency of the DNP process.^45^ New AMUPOL-based radicals with an ‘open’-ring conformation in the vicinity of the nitroxide groups were proposed, including HydrOPol that yields enhancements as high as 330 at 9.4 T and 100 K. These principles were then shown to transfer to the AsymPol family with the introduction of cAsymPol-POK and cAsymPol-TEK.^50,54^ Finally, it was recently shown that strong electron-nuclear hyperfine couplings and a proton-dense environment provide spin-diffusion pathways to rapidly transport hyperpolarization away from the biradical molecule into the bulk of the sample, leading to the development of the high-performance TEKPol derivative NaphPol.^52^ Venkatesh *et al.* reported a systematic evaluation of the overall sensitivity gains provided by a series of 18 dinitroxides at 9.4 T, concluding that a glass ceiling in DNP performance for CE dinitroxide biradicals might have been reached at this magnetic field.^71^ Most of these studies were carried in a relatively slow MAS regime (typically at 10 kHz) where depolarization losses^58,65,72^ are modest and the overall benefit in sensitivity for CE DNP^73^ remains high.

Despite this progress, transposing these developments from 9.4 T to the highest fields and fastest MAS frequencies available today is still a bottleneck. The electron-to-nucleus polarization transfer occurs through a series of adiabatic rotation-induced energy level crossings, the efficiency of which is sensitive to the magnetic field and the MAS frequency.^58,65^ Thus, as the breadth of the EPR profile of PAs scales with the magnetic field, the saturation of electron spin transitions by the microwave irradiation becomes less effective, and the CE event has a lower probability. In addition, depolarization losses^58,65,72^ increase at fast MAS for dinitroxides having medium-sized intramolecular magnetic couplings.^74^

Hybrid biradicals such as TEMtriPols,^34,51^ HyTEKs,^40^ NATriPols,^44^ SNAPols^47^ or PyrroTriPol^51^ recently appeared as promising PAs for DNP at high magnetic fields (≥18.8 T) and fast MAS. The narrow EPR line of one of the two radical units facilitates the saturation of the corresponding electron. In the case of HyTEK2, this combines with a strong electron–electron hyperfine coupling, giving a DNP efficiency that increases with the magnetic field increase.^40,75^ In parallel, efforts have also been devoted to refining the structure of dinitroxides so as to make them efficient at high-field and fast spinning, mostly by tailoring the strength of the intramolecular magnetic couplings. This led to the recently introduced TinyPol^46^ families of water-soluble dinitroxides, designed to have a relatively short linker and therefore sizeable electron–electron dipolar and *J*-exchange interactions. However, these radicals provide DNP efficiencies at 18.8 T that are still significantly lower than their analogs at 9.4 T.

Here, capitalizing on key design principles established for dinitroxides at intermediate magnetic fields, we introduce a new series of water-soluble dinitroxide biradicals with scaffolds augmented by dihydroxypropyl antenna chains that perform significantly better than previous dinitroxides at 18.8 T. We find that the new radical M-TinyPol(OH)_4_ yields enhancement factors of ∼220 at 18.8 T and 60 kHz MAS. The radicals are designed to improve the transfer of polarization away from the molecule and into the bulk, and this is validated by ^2^H ESEEM measurements to probe solvent accessibility supported by Molecular Dynamics simulations and by experiments on deuterated samples. This provides a rational understanding of why the dihydroxypropyl chains present in the best-performing structures are essential to deliver the polarization to the bulk solution.

## Results and discussion

### TinyPol structures

We recently introduced TinyPol and M-TinyPol, for DNP at 18.8 T.^46^ The good performance (*ε*_H_ ∼80–90) observed for these radicals was accounted for by an increase in the magnetic interactions between the two unpaired electrons. In this work, five new tailored TinyPol structures were prepared following updated design principles. Their structures, as well that of M-TinyPol^46^ and AsymPol-POK,^39^ are shown in Table 1. Details regarding the synthesis of the molecules are provided in the ESI.† The structure of TinyPol is presented in the ESI (Scheme 1†) as a reminder.

**Table 1:** Radicals investigated in this work, their DNP performance, and results of EPR measurements. Columns 1 and 2 show the names and the molecular structures respectively, columns 3 and 4 report respectively the proton MAS DNP enhancements and polarization build-up times. All the DNP data were measured in d_8_-glycerol/D_2_O/H_2_O 60/30/10 (v/v/v) at 18.8 T in 1.3 mm rotors, 105 ± 5 K and 40 kHz MAS, with a radical concentration of 10 mM. The sample temperature was carefully monitored with KBr and equilibrated for the microwave on and off measurements. Columns 6 and 7 show the electron spin relaxation parameters, *T*_ir_ and *T*_m_. The relaxation parameters were measured at W band at 105 K using 100 μM solutions in d_8_-glycerol/D_2_O/H_2_O 60/30/10 (v/v/v). Column 8 reports the solvent accessibility parameter extracted from EPR ESEEM experiments at 50 K using 200 μM solutions in d_8_-glycerol/D_2_O/H_2_O 60/30/10 (v/v/v) as detailed in the ESI.† Column 9 shows the weighted average of the exchange coupling ∣*J*∣, measured with room-temperature X-band EPR from a fitting procedure using EasySpin^76^ as detailed in the ESI†

Radical	Structure	DNP performance, 18.8 T, 40 kHz MAS				EPR parameters			
		*ε*_H_	*T*_B,ON_ (s)	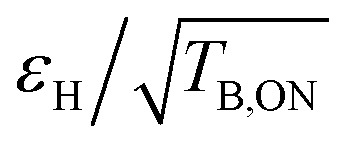	*ε*_DEPO_	*T*_ir_ (μs)	*T*_m_ (μs)	*Π*(D_2_O)	〈∣*J*∣〉 (MHz)
TinyPol-NH	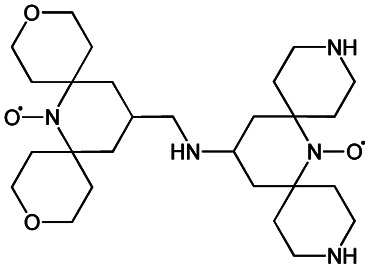	71 ± 4	25.4 ± 1	14.1	n.d.	306 ± 14	6.8 ± 0.4	0.37 ± 0.04	14.1
O-TinyPol	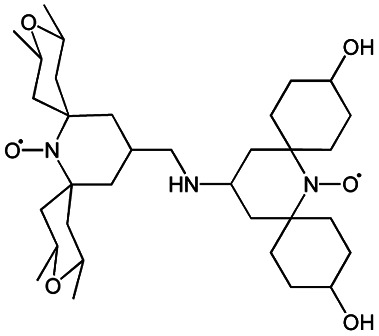	110 ± 5	9.3 ± 0.5	36.1	n.d.	284 ± 14	3.3 ± 0.4	0.37 ± 0.04	27.7
TinyPol(OH)_4_	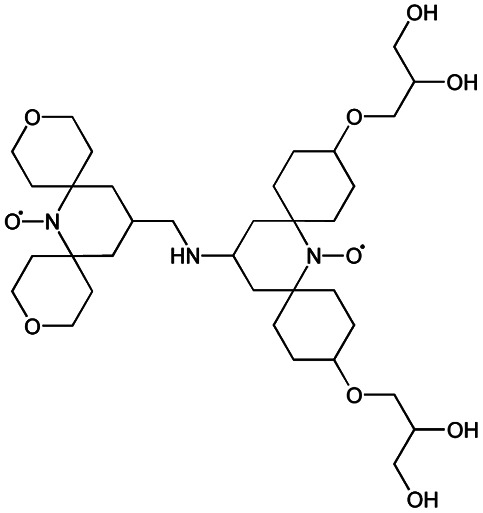	130 ± 6	8.9 ± 0.5	43.0	n.d.	250 ± 13	6.9 ± 0.4	0.34 ± 0.03	29.2
O-TinyPol(OH)_4_	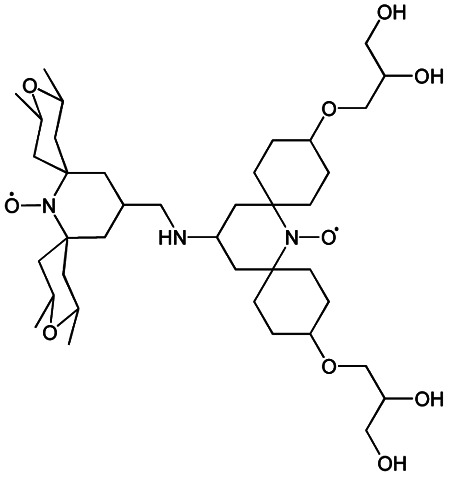	159 ± 6	8.3 ± 0.5	55.0	0.68	258 ± 13	2.8 ± 0.1	0.35 ± 0.03	27.1
M-TinyPol	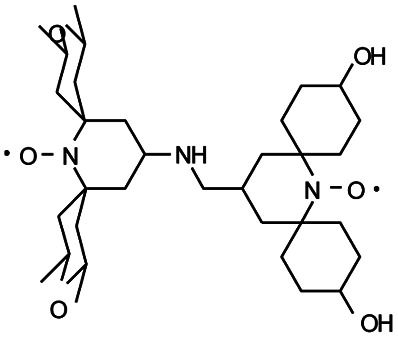	124 ± 6	16.8 ± 1	30.2	0.86	256 ± 14	1.9 ± 0.2	0.3 ± 0.04	25.3
M-TinyPol(OH)_4_, (M-TinyPol(OH)_4_)-d_10_)	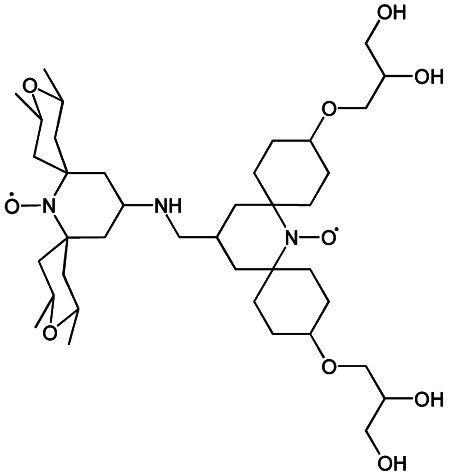	138 ± 7, (103 ± 5)	7.5 ± 0.5, (8.6 ± 0.4)	50.4, (35.1)	0.68	251 ± 13	2.9 ± 0.1	0.32 ± 0.03	27.5
AsymPol-POK	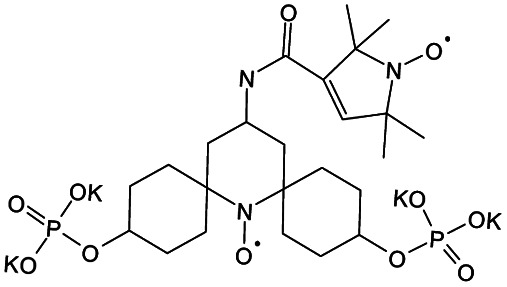	75 ± 7	4.4 ± 0.2	37.6	0.66	199 ± 10	<2.00	0.43 ± 0.04	80.5^a^

a This value is from ref. 39.

TinyPol-NH, O-TinyPol, TinyPol(OH)_4_ and O-TinyPol(OH)_4_ are based on the TinyPol scaffold while M-TinyPol(OH)_4_ is derived from M-TinyPol. The functionalization of the spirocyclohexyl rings with aliphatic chains bearing hydroxyl groups increases the molecular weight of TinyPol(OH)_4_, O-TinyPol(OH)_4_ and M-TinyPol(OH)_4_ structures as well as the hydrogen bonding interactions with the frozen matrix. This is first expected to lengthen the electron spin relaxation times, which is beneficial for their DNP performance. This design principle is well established since the development of bCTbK^31^ and TEKPol^30^ derivatives and is now embedded in most contemporary dinitroxide PAs.^50,52,54,71^ The local density of protons around the unpaired electron was also suggested to be a key parameter that modulates the DNP performance of dinitroxides at 9.4 T. Using a series of deuterated compounds, Venkatesh *et al.* recently demonstrated that the PA protons located beyond the cyclohexyl groups in TEKPoL play a key role in the DNP process,^52^ relaying the hyperpolarization outside of the spin diffusion barrier,^77^ as postulated by Perras *et al.* from simulations.^78^ Here, the chains decorating the cyclohexyl rings are potentially expected to play a similar role.

The local structure around the nitroxide, with open (O-) or closed (C-) conformations for the tetrahydropyran groups was also shown to significantly affect the efficiency of the DNP process in AMUPol, PyPol and PyTol derivatives,^45^ and this was tentatively correlated to the accessibility of solvent molecules to unpaired electrons. Fully open conformers were shown to outperform nitroxides with closed conformations. Locking the conformation of the tetrahydropyran rings in the O-form was achieved by introducing stereo-controlled *cis*-2,6 dimethyl groups.

This effect was then exploited in cAsymPolTEK where the “open” forms were again found to be significantly more efficient.^54^ Here, M-TinyPol and M-TinyPol(OH)_4_ have fully open conformations on one side of the molecule, as is the case for O-TinyPol(OH)_4_ which is the open (O-) conformer of TinyPol(OH)_4_.

In unsubstituted tetrahydropyran rings, various conformations including the O- and C- conformations co-exist. This is the case for the left side of TinyPol-NH and TinyPol(OH)_4_ molecules in Table 1. The functionalization of the cyclohexanol rings with the dihydroxylpropyl chains has no impact on the stereochemical constraints, and thus half-open and half-closed conformations are maintained.

We find such conformations in TinyPol(OH)_4_, O-TinyPol(OH)_4_, M-TinyPol and M-TinyPol(OH)_4_ in the right side of the structures according to schemes of Table 1. The DFT optimized structures of the six structures in the series are reported in Fig. S16.†

**Fig. 1:**
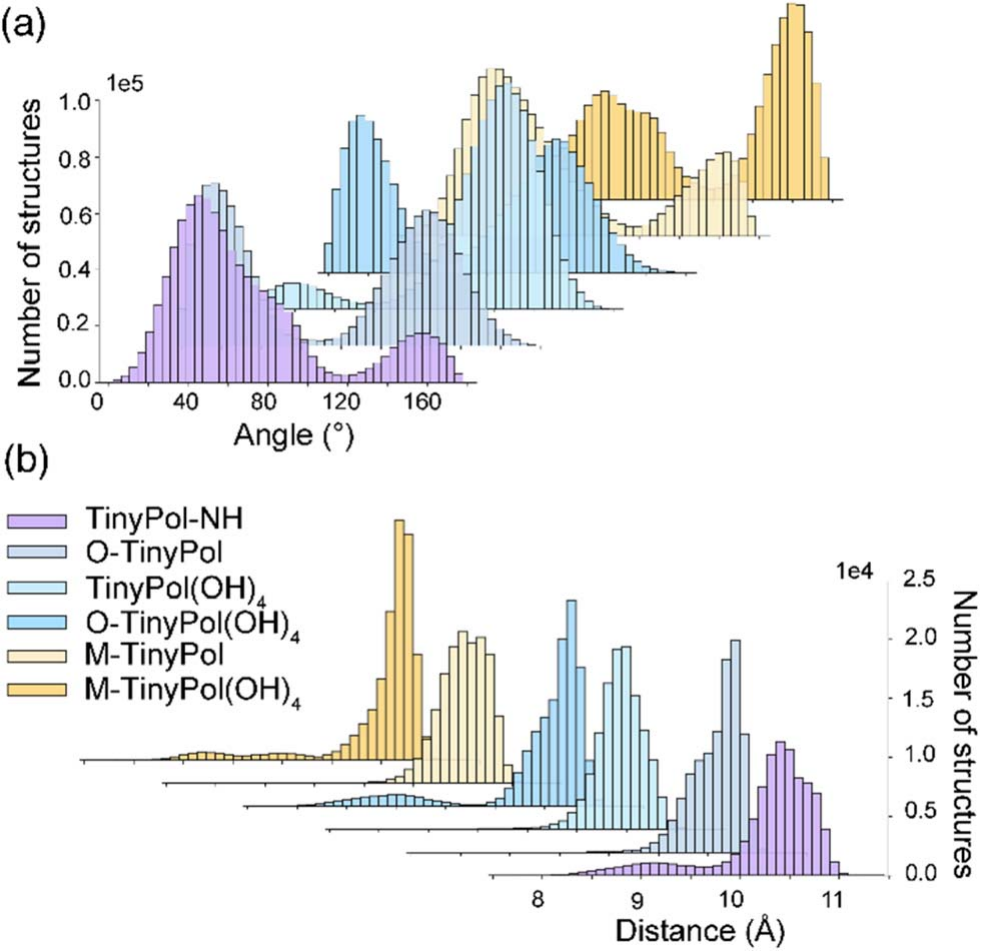
(a) and (b) Angle and distance distribution between the two unpaired electrons obtained from MD simulations. The distance was calculated by setting the position of the electron at the middle of the NO bond. The angle is between the two nitroxide planes. For each TinyPol structure, the results of 5 MD runs are reported separately in the ESI (Fig. S17 and S18†).

Molecular dynamics (MD) simulations were carried out to probe the conformational space of the PAs investigated here. Fig. 1(a) and (b) report respectively the distribution of angles between the two nitroxide planes and electron–electron distances. All TinyPols show one main e–e distance, between 10.4 and 10.8 Å, corresponding to an e–e dipolar coupling of ∼45 MHz. These dipolar couplings *D*, together with the exchange *J*-couplings drive the DNP cross-effect mechanism. MD trajectories highlight two main angles with broad distributions, at around 20° and 110–130° for O-TinyPol, TinyPol(OH)_4_ and O-TinyPol(OH)_4_, and three angles at around 50–60°, 80–100° and 120–160° for TinyPol-NH, M-TinyPol and M-TinyPol(OH)_4_. Individual plots are displayed in Fig. S17 and S18† for 5 MD runs.

### Electron relaxation properties

**Fig. 2:**
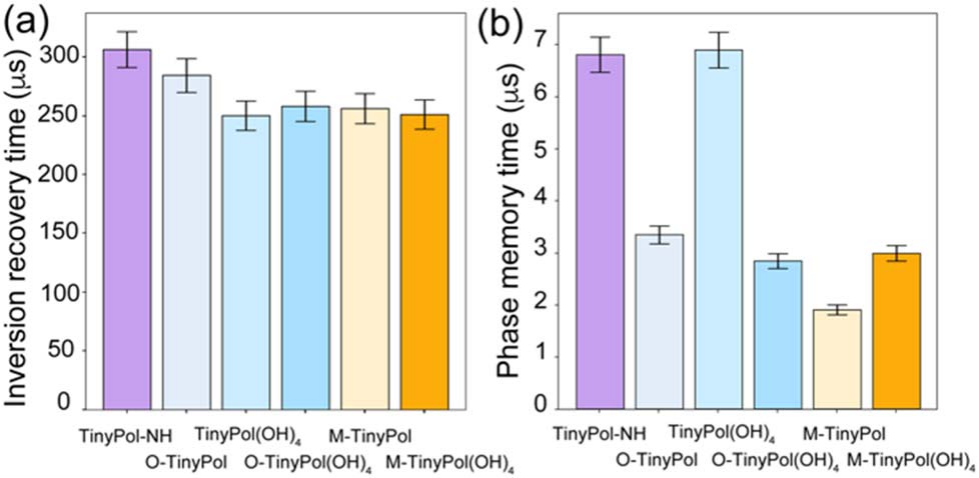
(a) Electron inversion recovery time (*T*_ir_) and (b) electron phase memory time (*T*_m_), for the six radicals investigated here. The electron relaxation parameters were measured at W band at 105 K using 100 μM solutions in d_8_-glycerol/D_2_O/H_2_O 60/30/10 (v/v/v).

Fig. 2 reports the measured electron relaxation times. The *T*_ir_ were found to be quite similar across the series, ranging between 250 and 306 μs, while the *T*_m_ vary between around 2 and 7 μs. The latter are shortened in biradicals presenting an open conformation on one side of the molecule, most likely due to the presence of the methyl groups.^30^ Due to the disparity in *T*_m_, the saturation factors (defined as the product of *T*_ir_ and *T*_m_) in turn range across an order of magnitude, from *ca.* 220 μs^2^ for M-TinyPol to *ca.* 2080 μs^2^ for TinyPol-NH as displayed in Fig. S15.†

Electron relaxation times were also measured for AsymPol-POK.^39^ Values significantly shorter than those of TinyPols were obtained as reported in Table 1, in line with expectations.^31^

### DNP performance

The DNP performance (enhancement *ε*_H_ and build-up times *T*_B,ON_) at 18.8 T and 40 kHz MAS of the TinyPol derivatives investigated here is summarized in Table 1 for 10 mM radical in d_8_-glycerol/D_2_O/H_2_O 60/30/10 (v/v/v). As a reference, experimental data are also reported for AsymPol-POK.

We also report in Table 1 the sensitivity factor 
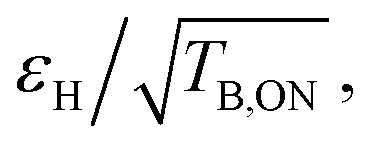
 which is a more relevant reporter of the overall DNP efficiency than *ε*_H_ alone,^63,65,73^ as short polarization build-up times can compensate for lower enhancements, and *vice versa*. Depolarization values are reported in Table S7† for some of the structures. We first observe that TinyPol-NH yields a relatively modest enhancement of ∼70, despite its large saturation factor as well as long polarization build-up time (>25 s). O-TinyPol, which is the open form of TinyPol, yields to a sizeable 110-fold enhancement factor. A further increase of *ε*_H_ is observed upon addition of dihydroxypropyl chains to the spirocyclohexyl groups, on one side of the radical, in place of the NH or OH groups for TinyPol(OH)_4_ and its open version O-TinyPol(OH)_4_. The build-up times for these three polarizing agents are significantly shorter than those of TinyPol-NH, reflecting a much faster polarization transfer rate to the bulk solution.^77^ In particular O-TinyPol(OH)_4_ has a relatively short DNP build-up time, which combined with *ε*_H_ ∼160 maximizes 
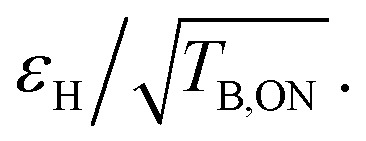
 Similarly, the addition of the dihydroxypropyl chains to the M-TinyPol scaffold leads to a higher enhancement factor. Thus, M-TinyPol(OH)_4_ reaches almost 140-fold enhancement factors at 40 kHz MAS. M-TinyPol(OH)_4_ also displays a significantly shorter *T*_B,ON_ than M-TinyPol. Both effects combine to yield a high 
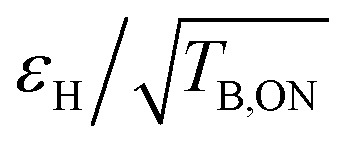
 value.

**Fig. 3:**
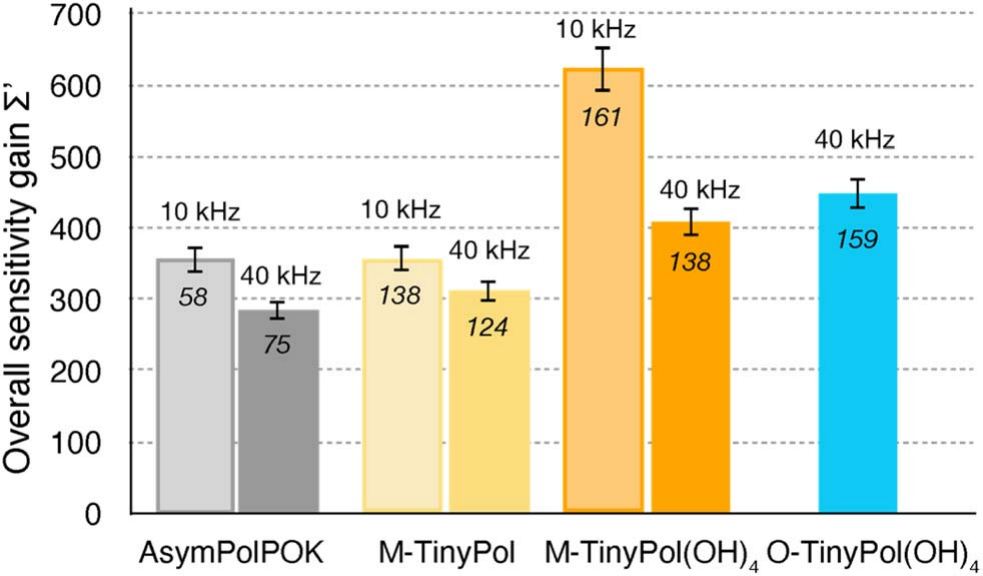
Overall sensitivity factors of 10 mM M-TinyPol, AsymPol-POK, M-TinyPol(OH)_4_ and O-TinyPol(OH)_4_ solutions in d_8_-glycerol/D_2_O/H_2_O 60/30/10 (v/v/v) acquired at 18.8 T in 1.3 mm zirconia rotors at 10 or 40 kHz MAS frequencies. The sample temperature was ∼110 K. The overall sensitivity factor is calculated as the product of *ε*_H_, the depolarization factor, and the square root of the ratio between the pure solvent *T*_1_ and the polarization build-up time *T*_B,ON_, as described in the ESI.† Notably, in this calculation, the paramagnetic quenching factor was not considered, while signal losses due to depolarization, as well as the value of *T*_B,ON_, were taken into account. Enhancement factors *ε*_H_ are indicated in italic.

In Fig. 3, overall sensitivity gains are calculated for O- and M-TinyPol(OH)_4_, and compared with the values measured for the reference M-TinyPol and AsymPol-POK radicals under similar experimental conditions. They were calculated from the proton enhancement *ε*_H_, scaled by the depolarization factor, and considering the gain of a shorter build-up time *T*_B,ON_ with respect to the pure solvent *T*_1_ (see ESI† for the detailed calculation of *Σ*′). M-TinyPol(OH)_4_ yields a gain of *ca.* 75% at 10 kHz MAS and 40% at 40 kHz MAS with respect to M-TinyPol. O-TinyPol(OH)_4_ also yields excellent performance at fast spinning frequencies.

While the superior performance of M-TinyPol(OH)_4_ over M-TinyPol could be tentatively ascribed to a higher saturation factor (or in other words to a better saturation of the electron spin transition due to a significantly longer *T*_m_, as shown in Fig. 2), such an argument does not explain the higher DNP efficiency of O-TinyPol(OH)_4_ with respect to TinyPol(OH)_4_. This suggests that the DNP performance of these dinitroxides is not only governed by their electron spin relaxation behavior, and that other parameters must be considered.

The relative orientation of the electron *g*-tensors could be one of these factors. Due to the flexibility of the linker, all TinyPol radicals investigated here display 2 to 3 main conformations, with distributions in the relative orientations of the nitroxide planes as reported by the MD simulations (Fig. 1(a)). Notably, only a small fraction of those distributions displays an optimal orientation with the two *g*-tensors in orthogonal planes.^28,66,79^ However, no correlation could be established between the distribution of conformations probed by MD at ambient temperature and the DNP performance. In contrast, we observe that M- and O-TinyPol(OH)_4_ have different distribution of *g*-tensor orientations but similar sensitivity factors 
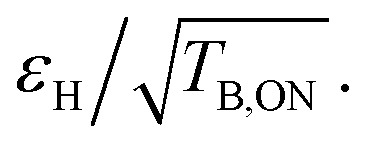
 Thus, while at cryogenic temperatures the distributions of conformations might be different, disparities in the relative orientation of the *g*-tensors does not appear to explain the differences observed in DNP performance within the TinyPol series.

The *ε*_H_ and *T*_B,ON_ values reported in Table 1 were measured in non-degassed solutions. However, paramagnetic oxygen (O_2_) dissolved within the DNP matrix, even at low concentration, is expected to act as effective relaxation sinks that will decrease the ^1^H polarization build-up time constant and in turn lead to a less efficient propagation of the hyperpolarization from the dinitroxides. Griffin and co-workers have recently showed that solid-effect enhancement of the water-soluble NMe_3_-BDPA radical in DNP juice, where build-up times were on the order of 60 s, could be improved by almost a factor 2 by degassing the sample.^80^ To evaluate if this effect is still observable for shorter build-up times, measurements were carried out for 10 mM O-TinyPol(OH)_4_ in both degassed and non-degassed solutions. The results (Fig. S21†) show only a moderated increase in the enhancement factors and polarization build-up times, leading to an increase in overall sensitivity of between 5 and 15%.

### Local proton density around the unpaired electron

To better understand the origin of the observed improved performances, we first turn our attention to the solvation of nitroxide moieties in the DNP matrix. The solvent accessibility was tentatively suggested to be a key parameter that modulates the density of protons around the unpaired electrons and in turn the DNP performance of dinitroxides at 9.4 T.^45^ This was based on the observation of a difference in solvent accessibility between fully open and fully closed conformations of the same radicals. To assess this effect, three pulse ESEEM experiments were conducted to probe proximities between the unpaired electron and the deuterated molecules in the matrix. The calculated solvent modulation depth *k*_D_ and solvent accessibility parameter *Π* (solvent) can be interpreted as an estimation of the number of deuterons present on a length scale in the range of 3 to 6 Å from the unpaired electrons, as the contribution of directly bonded nuclear spins is suppressed.^81^ All data are reported in Table S3† (and Fig. S11†), as well as details of the calculations of the *k*_D_ and *Π* parameters. Table 1 shows the extracted solvent accessibility *Π* parameters. All the TinyPol-like radicals here have similar accessibility to solvent molecules. This result is not surprising, considering the similar polarity around the unpaired electron and the available space nearby (the open or free conformations of the tetrahydropyran rings do not significantly reduce the access of solvent molecules, see DFT optimized structures of Fig. S16†).

**Fig. 4:**
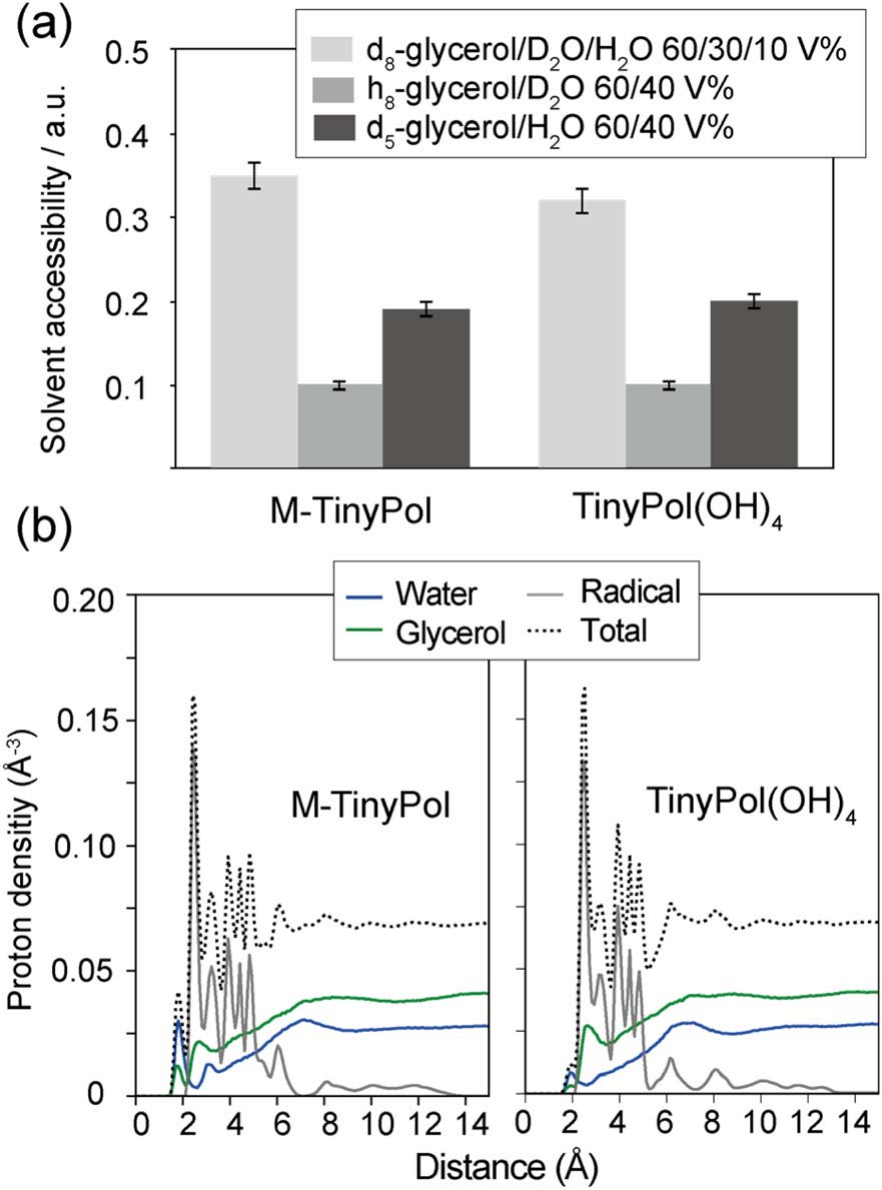
(a) Solvent accessibility parameters measured by X-band ESEEM experiments in 200 μM solutions of M-TinyPol and M-TinyPol(OH)_4_ in differently deuterated glycerol/water mixtures at 50 K. (b) Radial proton density distribution in a spherical range of 0 to 15 Å from the oxygen atom of the nitroxide group, calculated from MD simulations run in a glycerol/water 60/40 (v/v) mixture at 293 K as detailed in the ESI:† water protons (blue), glycerol protons (green), protons belonging to the biradical itself (grey).

In the light of recent work done by Griffin and co-workers,^82^ Bennati and co-workers^83^ as well as Stoll and co-workers,^84^ ESEEM experiments were conducted on solutions of M-TinyPol and TinyPol(OH)_4_, in either h_8_-glycerol/D_2_O 60/40 (v/v) or d_5_-glycerol/H_2_O 60/40 (v/v). Both of these solutions have *ca.* 40% ^2^H content, but with a different distribution, as almost all the deuterium atoms are on the water molecules in the first case while sitting on the non-exchangeable positions of glycerol in the second case. The results of these experiments are summarized in Fig. 4(a) and Table S4.†

We observed that the modulation depth and accessibility parameter are similar for M-TinyPol and TinyPol(OH)_4_ for all the three deuterated matrices tested, *i.e.* are independent on the biradical structure. However, both biradicals yield significantly different accessibility parameters using solvent mixtures with different deuterium distribution. More specifically, the ^2^H accessibility value almost doubles in d_5_-glycerol/H_2_O 60/40 compared to h_8_-glycerol/D_2_O 60/40 (v/v) (Fig. 4(a)). The enhanced modulation of the echo decay of the electrons, driven by distance-dependent hyperfine couplings, in d_5_-glycerol/H_2_O 60/40 (v/v) implies that there are more deuterons at a distance of 3 to 6 Å from the unpaired electrons in this solvent mixture. Similar observations were made for O-TinyPol(OH)_4_ (Fig. S12†). As deuterons are located only on glycerol (at non-exchangeable sites), this result points towards the predominance of glycerol deuterons at this 3 to 6 Å range, which matches roughly the size of the spin diffusion barrier.^77,82,85,86^ As expected, the solvent accessibility parameter is even higher when the d_8_-glycerol/D_2_O/H_2_O 60/30/10 (v/v/v) composition, the so-called “DNP juice”, is used, where glycerol molecules are fully deuterated, although susceptible to exchange with the 10% protonated water molecules. Overall, these data suggest that the shell volume surrounding the unpaired electrons, between 3 and 6 Å, is mostly occupied by deuterons from glycerol molecules, and this distribution is substantially similar for all TinyPol-like radicals.

To further interpret these results, the radial proton density in a sphere of 15 Å from the oxygen atom of the nitroxide group was calculated from 8001 snapshots from MD trajectories carried out in a glycerol/water 60/40 (v/v) mixture at 293 K. Calculations were done on both sides of the PA and average values were computed (as detailed in the ESI†). Fig. 4(b) shows the result of these calculations for M-TinyPol and TinyPol(OH)_4_. The plots for the other TinyPols in the series are presented in Fig. S20.† In both cases, these calculations indicate that, while the first solvation shell around the NO bond is mostly occupied by hydrogen-bonded water molecules with protons at about 2 Å distance from the oxygen atom, glycerol protons are predominant beyond this distance. Notably the glycerol molecules around the nitroxide yield a significant ^1^H density from 3 to 6 Å. This corroborates the ESEEM data.

Other recent investigations pointed out the key role of protons in the vicinity of the unpaired electrons, not only to allow an efficient CE DNP transfer through strong hyperfine couplings, but also to carry the hyperpolarization across the spin diffusion barrier away from the PA.

Notably, it was postulated^78^ and then demonstrated in TEKPoL that phenyl protons located in a 6 to 9.5 Å range from the unpaired electrons, are essential to transport the polarization into the bulk.^52^ Here, the ESEEM EPR data and the MD simulations show that deuterons from glycerol molecules, which unlike protons, cannot convey the ^1^H hyperpolarization across the spin diffusion barrier, are predominant in the second solvation shell of the nitroxide at a 2 to 4 Å range distance from the unpaired electron. Their presence may partly hinder the propagation of the hyperpolarization. In this regard, we hypothesize here that the hydroxypropyl chains in TinyPol(OH)_4_, O-TinyPol(OH)_4_ and M-TinyPol(OH)_4_ create pathways to relay the polarization to the bulk sample by proton spin-diffusion, in analogy to the aromatic antenna groups in NaphPol.^52^ Note that it was recently shown by simulations that in AsymPol series, radical protons in the vicinity of the unpaired electrons may not be necessary to relay the polarization.^87^

**Fig. 5:**
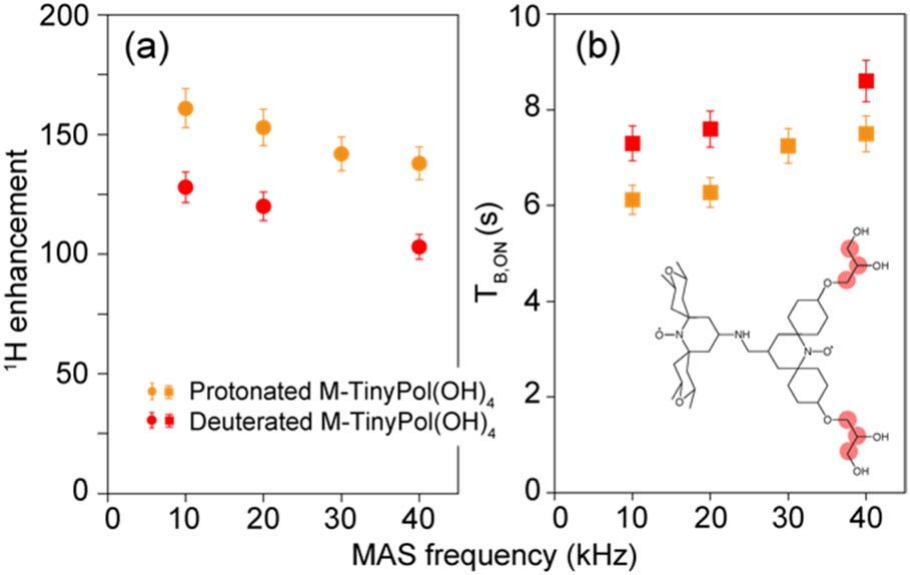
(a) Enhancement factors and (b) polarization build-up times of protonated and deuterated M-TinyPol(OH)_4_ at a 10 mM concentration in bulk solutions of d_8_-glycerol/D_2_O/H_2_O 60/30/10 (v/v/v), as a function of MAS frequency. Enhancements values were measured at 18.8 T from proton NMR spectra in 1.3 mm zirconia rotors at a sample temperature of 110 ± 5 K. Experimental details are given in the ESI.† The position of the deuterons are indicated by pale red circles in the molecular structure of M-TinyPol(OH)_4_.

To validate this hypothesis, a deuterated version of M-TinyPol(OH)_4_ was prepared, in which the protons of the hydroxypropyl chains were replaced by deuterons. Fig. 5 compares the enhancement and build-up times in protonated and deuterated M-TinyPol(OH)_4_ as a function of the spinning frequency. Deuteration of the side chains leads to lower enhancement factors and longer build-up times. This observation is in line with what was observed for a series of deuterated TEKPoL PAs, where ^1^H DNP enhancements were shown to decrease with higher biradical deuteration levels while the DNP build-up times concomitantly increased, almost all the deuterated forms having lower *ε*_H_ and longer *T*_B,ON_ than the protonated radical.^52^ This also confirms that the protons in the chains provide new channels to propagate the hyperpolarization to the bulk, resulting in superior overall DNP performance, *i.e.* shorter polarization build-up time or higher polarization transfer coefficient at the spin diffusion barrier interface *k*_DNP_, according to the model of Prisco and co-workers.^77^

We note that the 
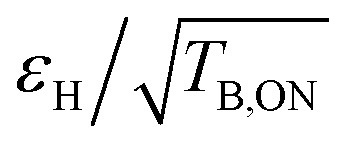
 factor drops from 50 to 35 upon deuteration, *i.e.* the overall sensitivity of deuterated M-TinyPol(OH)_4_ almost returns to that of M-TinyPol (Table 1), which differs from M-TinyPol(OH)_4_ only by the absence of the hydroxypropyl chains.

### Performance of M-TinyPol(OH)_4_ at very fast MAS

**Fig. 6:**
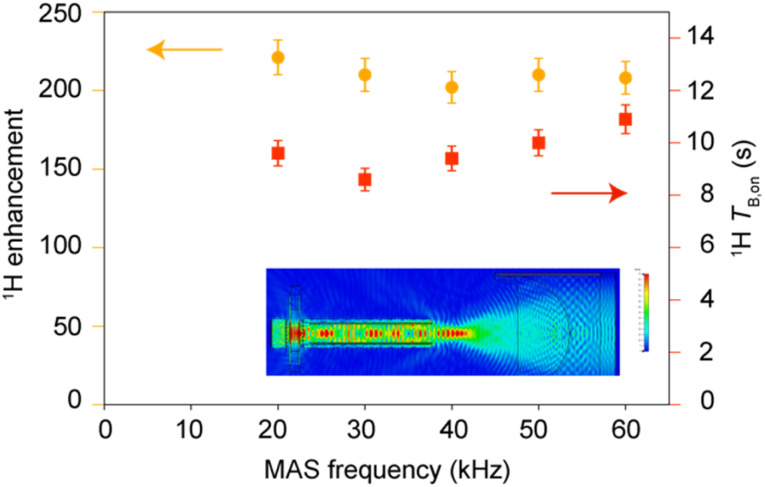
Enhancement factors and polarization build-up times of 10 mM M-TinyPol(OH)_4_ in a bulk solution of d_8_-glycerol/D_2_O/H_2_O 60/30/10 (v/v/v), as a function of MAS frequency. Enhancements values were measured at 18.8 T from proton NMR spectra, in a 0.7 mm zirconia rotor at a sample temperature of 110 ± 5 K. The inset shows the microwave distribution within the rotor predicted by finite-element simulations.

The performance of M-TinyPol(OH)_4_ at MAS rates up to 60 kHz measured in a 0.7 mm DNP probe is shown in Fig. 6. The predicted μ-wave distribution within the rotor was calculated from finite-element simulations and is displayed as an inset in Fig. 6. The average microwave field is calculated to be slightly higher than in 1.3 mm rotors 1.76 *versus* 1.07 MHz//W^−1/2^). Here, we note that there are several reasons for the superior μ-wave performance of the 0.7 mm probe. First, the portion of the sample volume that is irradiated by the beam is about 10% larger compared to that of a 1.3 mm rotor (Fig. S23†). Furthermore, the beam transmitted through the probe's waveguide is focused to a smaller diameter thereby increasing the intensity of the microwave B_1_ field. While the thicker zirconia wall of the 1.3 mm rotor also introduces more losses, its impact on the B_1_ field strongly depends on the wall thickness-to-wavelength ratio due constructive *versus* destructive interference, as detailed in the ESI.† Enhancement factors of between 204 and 214 were measured over the whole spinning frequency range, with build-up times ranging from 8.5 to 11 s. These values are the highest reported so far for dinitroxides at high fields and fast MAS frequencies, highlighting the importance of the instrumentation and of efficient microwave penetration to obtain high signal amplification.^88,89^

## Conclusion

In summary, a series of new TinyPol radicals were synthesized and their DNP performance was examined at 18.8 T, 100 K and 40 kHz MAS. We find that the introduction of protonated hydroxypropyl chains decorating the spirocyclohexyl groups around the nitroxide rings significantly improves the DNP efficiency of TinyPol radicals, both in terms of enhancement factors, ^1^H DNP build-up times and overall sensitivity gain. Combining this novel structural element with a nitroxide having an open conformation of the tetrahydropyran rings results in O-TinyPol(OH)_4_ and M-TinyPol(OH)_4_ that provide a sensitivity gain almost twice that of previously reported dinitroxides.

We find that deuteration of the hydroxypropyl chains decreases enhancement factors while increasing build-up times, suggesting that protons located in the chains (*i.e.* close to but not in the immediate vicinity of the electrons) are key to transporting polarization across the spin diffusion barrier into the bulk.^52,77^ This analysis is supported with ^2^H ESEEM measurements and MD simulations that suggest that the deuterated glycerol molecules of the DNP matrix are located mainly in the second solvation shell of the NO bond, limiting access to protonated water molecules. Overall, this provides a rational framework for why protons in the chains are important to delivering the polarization to the bulk solution, and provides a clear guideline for the future developments of new polarizing agents but also of optimized formulations.

## Author contributions

L. N., G. M., S.-E. A., D. G., A. V., Z. W., T. R. conducted and analysed DNP NMR experiments. L. N. developed and conducted the molecular dynamic simulations. L. N and D. S. ran the DFT calculations. M. Y., L. N. and G. M. conducted EPR measurements. G. C. and O. O. synthesised the radicals. C. R. and A. P. did the finite-element simulations. A. L., M. L. and O. O. conceptualized the research in close consultation with L. E. and acquired necessary funding. All authors participated in the analysis and discussion of the experiments, and related results. The manuscript was written through contributions of all authors. All authors have given approval to the final version of the manuscript.

## Conflicts of interest

There are no conflicts to declare.

## Supplementary Material

SC-015-D4SC04473H-s001

## Data Availability

Data that support the findings of this study are available from the corresponding author upon reasonable request. The data supporting this article have been included as part of the ESI.†
