# Supplementary material for: Efficient DNP at high fields and fast MAS with antenna-sensitized dinitroxides
Source: Chem Sci. 2024 Sep 12;15(40):16582–93. doi: 10.1039/d4sc04473h (PMC11411413; doi:10.1039/d4sc04473h)
Supplement: SC-015-D4SC04473H-s001 [file SC-015-D4SC04473H-s001.pdf]

Supplementary Information (SI) for Chemical Science.
This journal is © The Royal Society of Chemistry 2024

Supporting Information

Efficient DNP at High Fields and Fast MAS with Antenna-Sensitized Dinitroxides

Lorenzo Niccoli,†[a][b][c][d] Gilles Casano, †[e] Georges Menzildjian,†[a] Maxim Yulikov,[f] Thomas Robinson,[a] Salah-Eddine Akrial,[a] Zhuoran Wang,[a] Christian Reiter,[g] Armin Purea,[g] Didier Siri,[e] Amrit Venkatesh,[h][i] Lyndon Emsley,[h] David Gajan,[a] Moreno Lelli,* [b][c][d] Olivier Ouari,*[e] and Anne Lesage*[a]

[a] *Centre de RMN à Hauts Champs de Lyon, UMR 5082, Université de Lyon (CNRS/ENS Lyon/UCBL),5 rue de la Doua, Villeurbanne, 69100, France*
[b] *Center of Magnetic Resonance (CERM), University of Florence, 50019, Sesto Fiorentino, Italy*
[c] *Department of Chemistry 'Ugo Schiff', University of Florence, Via della Lastruccia 13, 50019 Sesto Fiorentino, FI, Italy.*
[d] *Consorzio Interuniversitario Risonanze Magnetiche Metalloproteine Paramagnetiche (CIRMMP), Via Luigi Sacconi 6, 50019 Sesto Fiorentino, FI, Italy*
[e] *Aix Marseille Uni, CNRS, ICR, 13013 Marseille, France*
[f] *Department of Chemistry and Applied Biosciences, Eidgenössische Technische Hochschule Zürich, CH-8093 Zürich, Switzerland*
[g] *Bruker Biospin, 76275 Ettlingen, Germany*
[h] *Laboratory of Magnetic Resonance, Institut des Sciences et Ingénierie Chimiques, École Polytechnique Fédérale de Lausanne (EPFL), CH-1015 Lausanne, Switzerland*

TABLE OF CONTENTS

S1 Synthesis details of the new series of TinyPol radicals and stability 3
S2 NMR experiments 12
S3 Continuous wave EPR 13
S4 ESEEM EPR experiments. 17
S5 Electron spin T_{ir} and T_m measurements 20
S6 Depolarization factor and overall sensitivity gain 24
S7 DFT geometry optimization 25
S8 Molecular Dynamics Simulations 26
S9 Proton density distribution around radical 29
S10 DNP performance in degassed solutions 30
S10 Finite element simulations 34
S11 References 35

S1 Synthesis details of the new series of TinyPol radicals and stability

Experimental: Compounds **1**, **4**, **7**, **8**, **15** and **17** were prepared according to the literature procedure.[1] All chemicals used in synthesis were purchased from Aldrich Chemical Co. Purification of products was accomplished by flash chromatography on silica gel (Merck silica gel 60, 230-400 mesh). NMR spectra were recorded on a Bruker AVL 300 spectrometer (^{1}H NMR 300.1 MHz and ^{13}C NMR 75.5 MHz) using $CDCl_3$ as the solvent (internal reference). Mass spectral analyses were carried out using a Q-STAR Elite at the Aix-Marseille University Mass Spectrum Facility, Spectropole Saint-Jérôme. EPR measurements were performed on a Bruker Elexsys spectrometer operating at 9.4 GHz (X-band) in 50 µL capillaries using the following parameters: microwave power 5 mW and modulation amplitude 0.4 G. The final products were purified to ≥95% and were confirmed by HPLC-MS analysis. HPLC-MS experiments were performed using an Agilent 1260 infinity system coupled with a 6120 simple quadruple. This system was equipped with a C12 column (Zorbax 1.8 µM, 3 x 50 mm) that was equilibrated with 10% vol. acetonitrile in 0.1% vol. formic acid aqueous solution at the flow rate of 0.21 mL/min.

S1.1. Synthesis of TinyPol(OH)$_4$ (3).

The TinyPol(OH)$_4$ series was synthesized according a two steps reaction starting from the corresponding TinyPol. First the epoxide was introduced by reaction with epichlorohydrin and the epoxide group was opened by hydrolysis in aqueous basic conditions, following a modified literature procedure.[2]

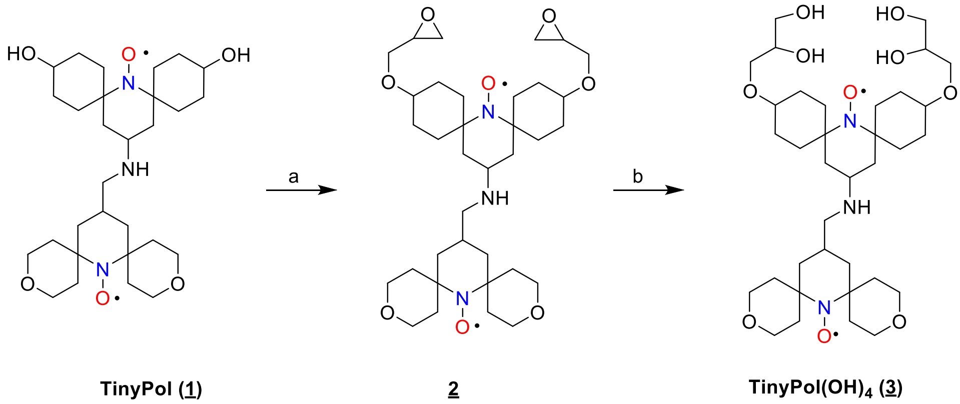

Scheme 1: Synthetic route to **TinyPol(OH)$_4$ (3).**

Reagents and conditions*: (a) Epichlorohydrin, Tetrabutylammonium hydrogensulfate, NaOH (50%) in H_2O, THF, 25°C, 48h. (b) KOH (1M), H_2O, THF, 60°C, 16h).*

Synthesis of compound 2.

To a stirred solution of 50% aqueous NaOH (10 mL) and tetrabutylammonium hydrogensulfate (100 mg, 0.3 mmol) was added compound **1** (0.23 g, 0.43 mmol) dissolved in THF/H_2O (2 mL/5 mL). At 0°C, epichlorohydrin (2.0 mL, 21.5 mmol) was added and the solution was stirred 12h. After this time, epichlorohydrin (2.0 mL, 21.5 mmol) was added and the solution was stirred during 12h. Then, the mixture was extracted twice with CH_2Cl_2, dried over Na_2SO_4 and concentrated under reduced pressure. The residue was purified by SiO_2 column chromatography using DCM/EtOH (97/3) as eluant to give compound **2** (0.1 g, 36%) as a red solid.

ESI-MS = 648.4 [M+H]$^+$; 670.4 [M+Na]$^+$; 686.4 [M+K]$^+$.

HPLC on C18 column (Zorbax 1.8 µM, 3 x 50 mm, flow rate of 0.21 mL/min). Retention time: 7.21 min.

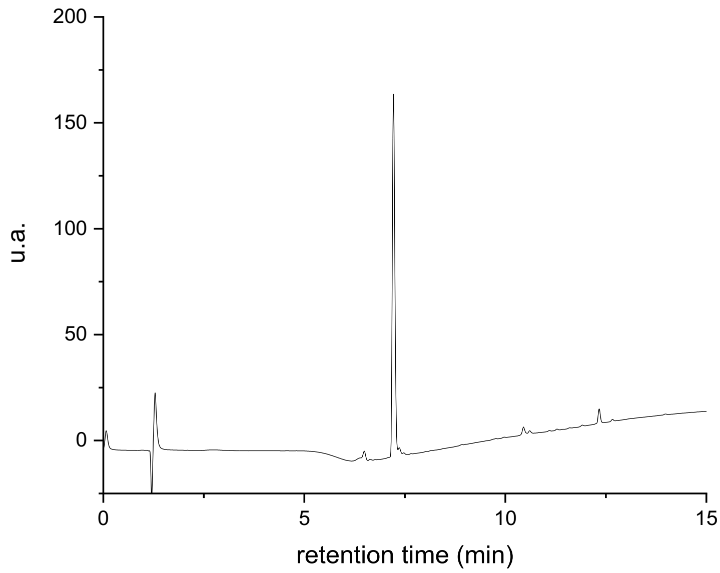

Figure S1: HPLC chromatogram of compound **2** (Water/ Acetontitrile/0.1% Formic Acid gradient, RP C18, UV detection at 254 nm).

Synthesis of compound 3.

To a stirred solution of compound **2** (23 mg, 0.035 mmol) in THF (0.3 mL) was added 2 mL of aqueous KOH (1M) solution and the mixture was stirred at 60°C overnight. After cooling, the solution was concentrated under reduced pressure. The residue was purified by SiO_2 column chromatography using DCM/EtOH (8/2) as eluant to give **TinyPol(OH)$_4$** (11 mg, 46%) as a pale orange solid.

HRMS-ESI: m/z: calcd for $C_{35}H_{62}N_3O_{10}^{2\cdot+}$ ($[M+H]^+$) 684.4430 found 684.4425.

HPLC on C18 column (Zorbax 1.8 µM, 3 x 50 mm, flow rate of 0.21 mL/min). Retention time: 5.99 min.

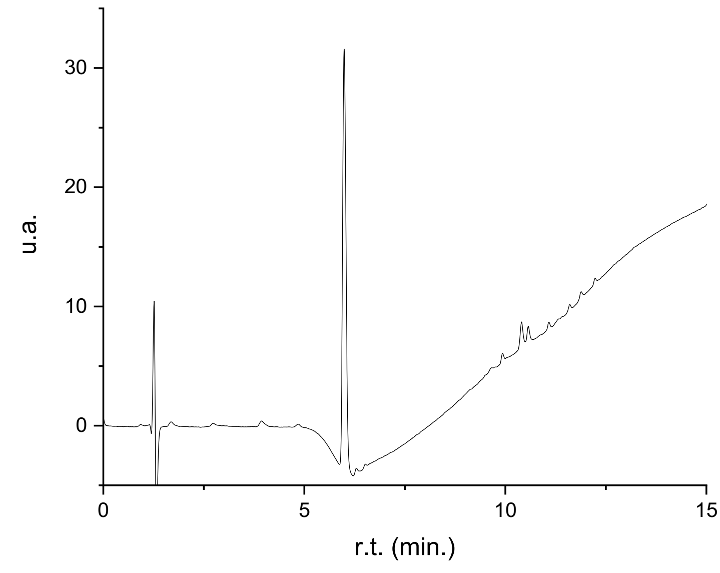

Figure S2: HPLC chromatogram of **TinyPol(OH)4** (Water/ Acetontitrile/0.1% Formic Acid gradient, RP C18, UV detection at 254 nm).

S1.2. Synthesis of M-TinyPol(OH)4 (6).

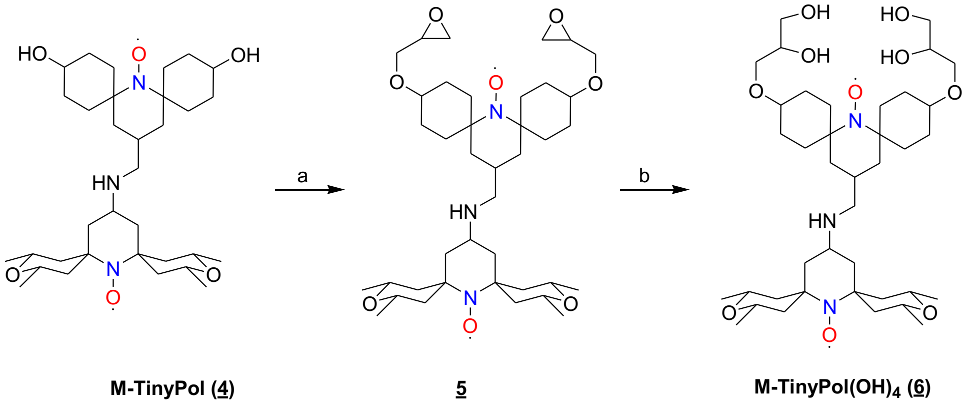

Scheme 2: Synthetic route to **M-TinyPol(OH)4 (6).**

Reagents and conditions: *(a) Epichlorohydrin, Tetrabutylammonium hydrogensulfate, NaOH (50%) in H_2O, THF, 25°C, 48h. (b) KOH (1M), H_2O, THF, 60°C, 6h.*

Synthesis of compound 5.

A total of 90 mg (30 %) of compound **5** was obtained as a red solid using the experimental procedure described for compound **2** starting with 250 mg of compound **4**.

HRMS-ESI: m/z: calcd for $C_{39}H_{65}N_3O_8Na^{2\cdot+}$ ($[M+Na]^+$) 726.4664 found 726.4666.

HPLC on C18 column (Zorbax 1.8 µM, 3 x 50 mm, flow rate of 0.21 mL/min). Retention time: 7.80 min.

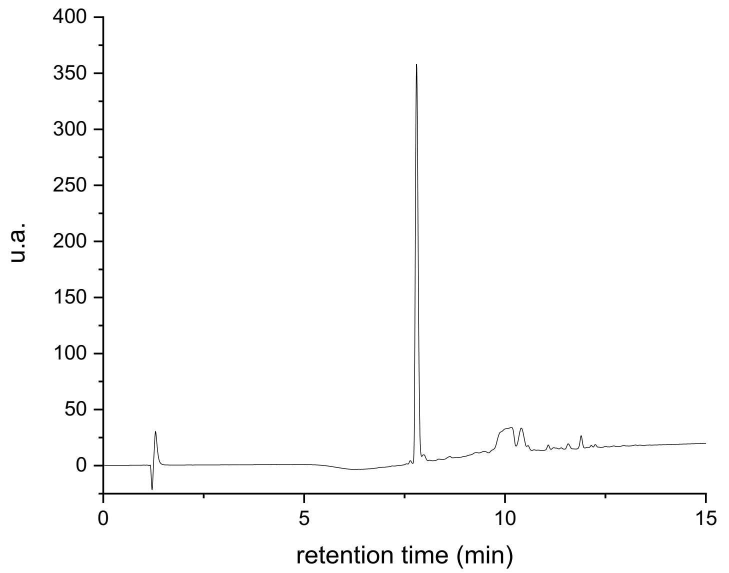

Figure S3: HPLC chromatogram of compound 5 (Water/ Acetonitrile/0.1% Formic Acid gradient, RP C18, UV detection at 254 nm).

Synthesis of compound <u>6</u>

Compound **6** (7.5 mg, 36%) was prepared according to the general procedure described for compound **<u>3</u>** starting with compound **<u>5</u>** (20 mg, 0.028 mmol).

HRMS-ESI: m/z: calcd. for $C_{39}H_{69}N_3O_{10}Na^{2\cdot+}$ ([M+Na]$^+$) 762.4875 found 762.4876.

HPLC on C18 column (Zorbax 1.8 µM, 3 x 50 mm, flow rate of 0.21 mL/min). Retention time: 7.06 min.

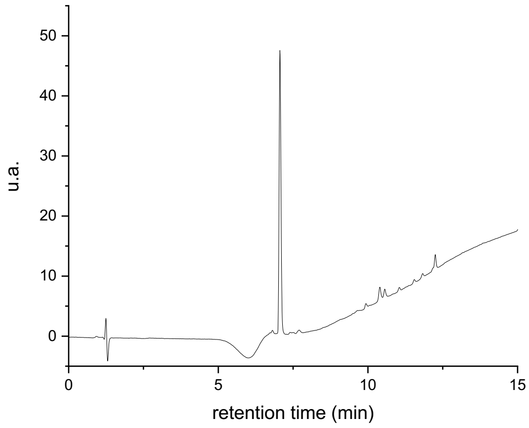

Figure S4: HPLC chromatogram of **M-TinyPol(OH)$_4$** (Water/ Acetontitrile/0.1% Formic Acid gradient, RP C18, UV detection at 254 nm).

S1.3. Synthesis of O-TinyPol (<u>9</u>) and O-TinyPol(OH)$_4$ (<u>11</u>).

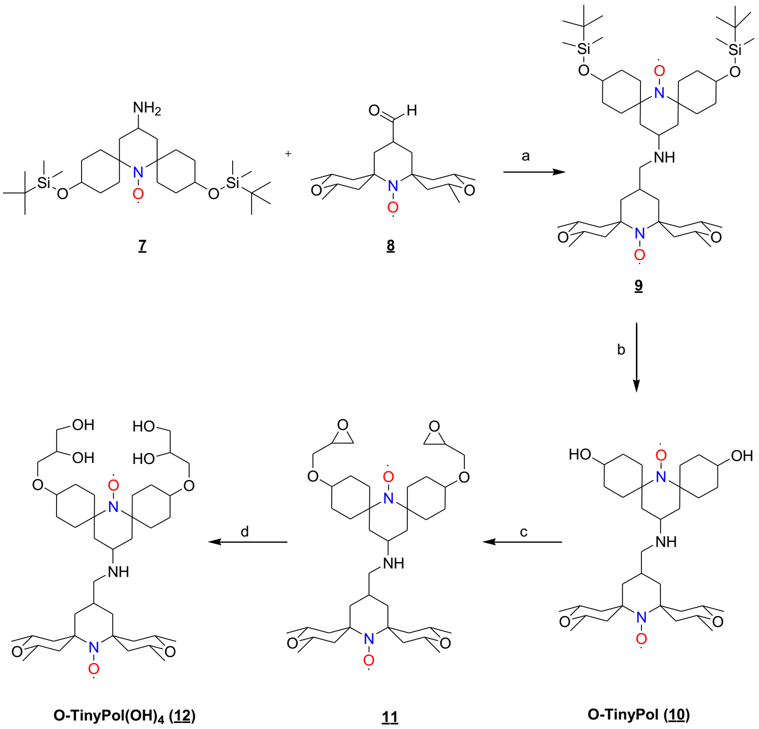

<u>**Scheme 3**</u>**:** Synthetic route to **O-TinyPol (<u>10</u>) and O-TinyPol(OH)$_4$ (<u>12</u>).**

Reagents and conditions: *(a) NaBH(OAc)$_3$, AcOH, THF, 25°C, 16h. (b) TBAF, THF, 25°c, 48h.(c) Epichlorohydrin, Tetrabutylammonium hydrogensulfate, NaOH (50%) in H_2O, THF, 25°C, 48h. (d) KOH (1M), H_2O, THF, 40°C, 16h.*

Synthesis of compound 9.

Compounds **7** (0.34 g, 0.66 mmol) and **8** (0.18 g, 0.55 mmol) were dissolved in THF (20 mL) under argon atmosphere. Then, AcOH was added to adjust pH around 7 and the solution was stirred at 25°C. $NaBH(OAc)_3$ (0.19 g, 0.89 mmol) was added in one portion at 0°C and the mixture was stirred overnight at 25°C. At the end of reaction, a saturated aqueous $NaHCO_3$ solution (50 mL) was added, the mixture was extracted two times with DCM (2 x 80 mL), dried over Na_2SO_4, concentrated under reduced pressure and the residue was purified by SiO_2 column chromatography using Pentane/AcOEt (1:1) to afford compound **9** (0.2 g, 44%) as an orange solid.
ESI-MS = 821 $[M+H]^+$; 411 $[M+2H]^{2+}$.

Synthesis of O-TinyPol 10.

Compound **9** (0.2 g, 0.24 mmol) was dissolved in dry THF (10 mL) under argon atmosphere. A 1M solution of TBAF in THF (3.3 mL) was added and the solution was stirred 24h at 25°C. After this time, the solution was concentrated under reduced pressure and the residue was purified by SiO_2 column chromatography using DCM / EtOH (8:2) to afford compound **10** (0.09 g, 63%) as a red solid.
HRMS-ESI: m/z: calcd for $C_{33}H_{57}N_3O_6Na^{2.+}$ ($[M+Na]^+$) 614.4140 found 614.4139.
HPLC on C18 column (Zorbax 1.8 μM, 3 x 50 mm, flow rate of 0.21 mL/min). Retention time: 6.788 min.

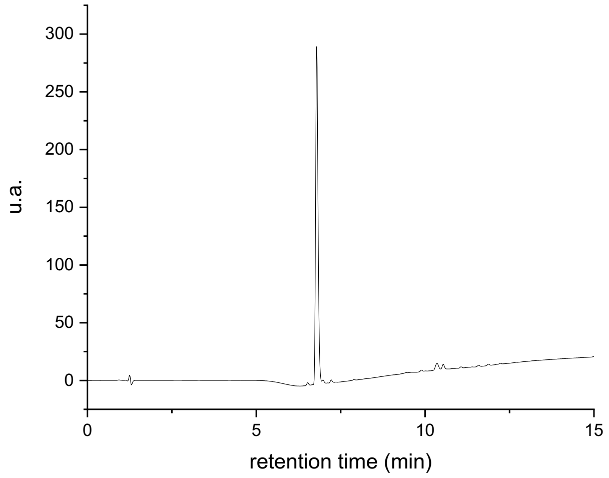

Figure S5: HPLC chromatogram of **O-TinyPol** (Water/ Acetontitrile/0.1% Formic Acid gradient, RP C18, UV detection at 254 nm).

Synthesis of compound 11.

A total of 18 mg (25%) of compound **11** was obtained as a red solid using the experimental procedure described for compound **2** starting with 60 mg of compound **10**.
ESI-MS = 704 $[M+H]^+$.

S1.4. Synthesis of O-TinyPol(OH)$_4$ (12).

Compound **12** (6.3 mg, 35%) was prepared according to the general procedure described for compound **3** starting with compound **11** (17 mg, 0.024 mmol).
HRMS-ESI: m/z: calcd for $C_{39}H_{69}N_3O_{10}Na^{2.+}$ ($[M+Na]^+$) 762.4875 found 762.4888.
HPLC on C18 column (Zorbax 1.8 μM, 3 x 50 mm, flow rate of 0.21 mL/min). Retention time: 6.755 min.

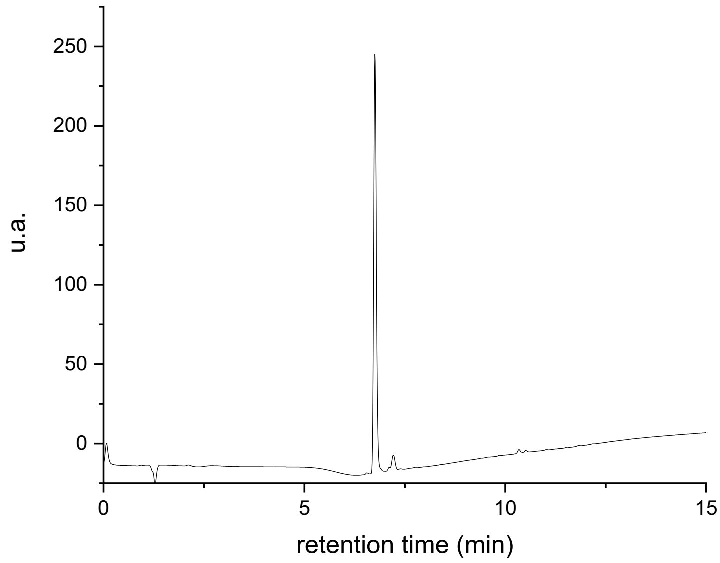

Figure S6: HPLC chromatogram of **O-TinyPol(OH)$_4$** (Water/ Acetontitrile/0.1% Formic Acid gradient, RP C18, UV detection at 254 nm).

S1.5. Synthesis of M-TinyPol(OH)$_4$-d$_{10}$ (14).

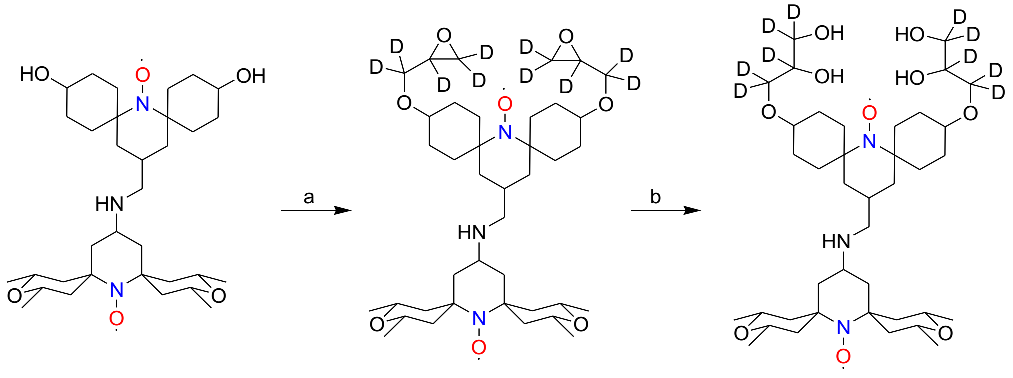

M-TinyPol (4) **13** **M-TinyPol(OH)$_4$D$_{10}$(14)**

Scheme 4: Synthetic route to **M-TinyPol(OH)$_4$-d$_{10}$ (14)**.
Reagents and conditions: *(a) Epichlorohydrin-d$_5$, Tetrabutylammonium hydrogensulfate, NaOH (50%) in H_2O, THF, 25°C, 48h. (b) KOD (1N), D_2O, THF, 60°C, 6h.*

Synthesis of compound 13.
To a stirred solution of 50% aqueous NaOH (5 mL) and tetrabutylammonium hydrogensulfate (50 mg, 0.15 mmol) was added compound **4** (0.12 g, 0.20 mmol) dissolved in THF/D_2O (1 mL/3 mL). Epichlorohydrin-*d$_5$* (1.0 mL, 10.7 mmol) was added and the solution was stirred 12h. After this time, epichlorohydrin (1.0 mL, 10.7 mmol) was added and the mixture was stirred during 12h. Then, the mixture was extracted twice with CH_2Cl_2, dried over Na_2SO_4 and concentrated under reduced pressure. The residue was purified by SiO_2 column chromatography using CH_2Cl_2/EtOH (97/3) as eluant to give compound **2** (35 mg, 25%) as a red solid.
ESI-MS= 714.5 $[M+H]^+$; 736.5 $[M+Na]^+$; 1428.1 $[2M+H]^+$; 1450.1 $[2M+Na]^+$.

HPLC on C18 column (Zorbax 1.8 µM, 3 x 50 mm, flow rate of 0.21 mL/min). Retention time: 7.67 min.

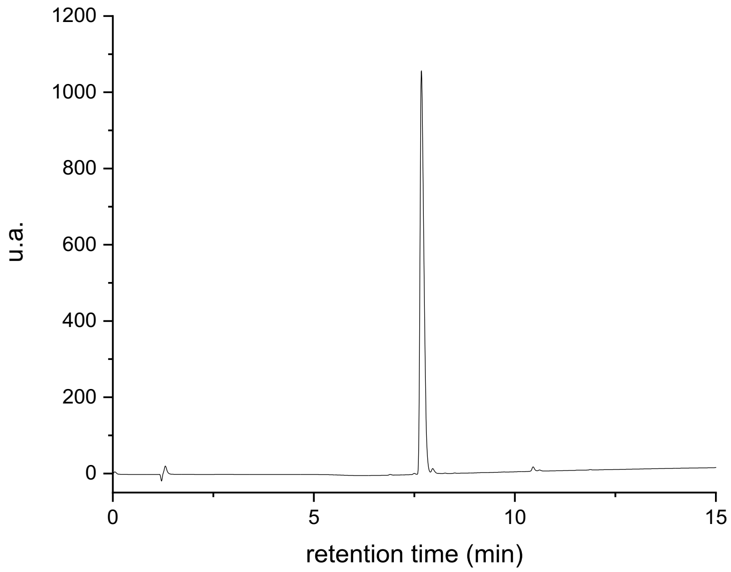

Figure S7: HPLC chromatogram of compound **13** (Water/ Acetontitrile/0.1% Formic Acid gradient, RP C18, UV detection at 254 nm).

Synthesis of compound 14.

To a stirred solution of compound **13** (35 mg, 0.049 mmol) in THF (0.5 mL) was added 2 mL of aqueous KOD (1M) solution and the mixture was stirred at 40°C overnight. After cooling, the solution was concentrated under reduced pressure. The residue was purified by SiO_2 column chromatography using CH_2Cl_2/EtOH (8/2) as eluant to give a red solid. The solid was dissolved in a large volume of distillate water (30 mL) and it was stirred for 2h under argon atmosphere. Then, the water was removed giving compound 14 (12.4 mg, 34%) as a red solid.

HRMS-ESI: m/z: calcd for $C_{39}H_{59}D_{10}N_3O_{10}Na^{2\cdot +}$ ([M+Na]$^+$) 772.5503 found 772.5504.

HPLC on C18 column (Zorbax 1.8 µM, 3 x 50 mm, flow rate of 0.21 mL/min). Retention time: 7.05 min.

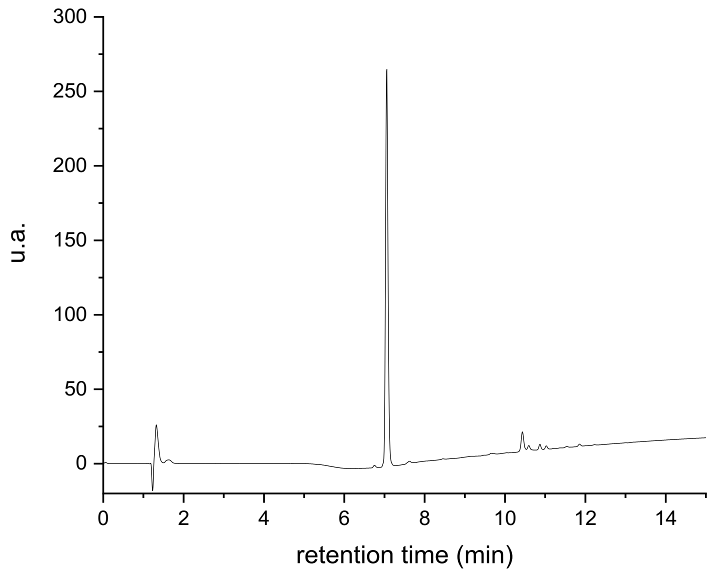

Figure S8: HPLC chromatogram of **M-TinyPol(OH)$_4$-d$_{10}$** (Water/ Acetontitrile/0.1% Formic Acid gradient, RP C18, UV detection at 254 nm).

S1.5. Synthesis of TinyPol-NH (19).

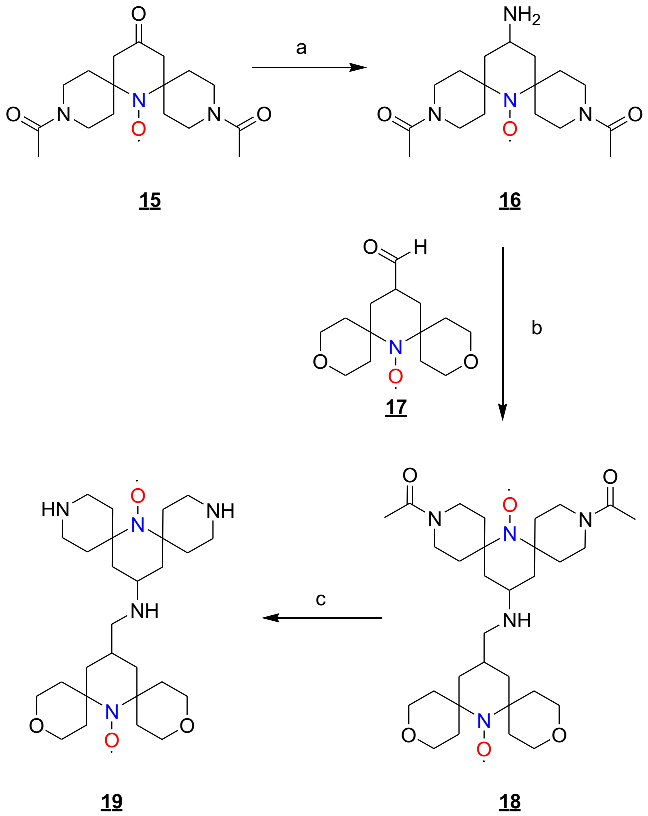

Scheme 5: Reagents and conditions used here. (a) NH_4OAc, $NaBH_3CN$, THF/EtOH, 30°C, 2h. (b) **17**, $NaBH(OAc)_3$, AcOH, DCM, 25°C, 4h. (c) KOH (6M), EtOH/H_2O, 80°C, overnight.

Synthesis of compound <u>16</u>.

Compound **<u>15</u>** (0.51 g, 1.51 mmol) was dissolved in THF/EtOH. (10 mL/10 mL) under argon atmosphere. NH_4OAc (1.16 g, 15.06 mmol) was added, and the mixture was stirred 2h at 30°C. After cooling, $NaBH_3CN$ (66 mg, 1.04 mmol) was added, and the solution was stirred overnight at 30°C. At the end of reaction, a saturated aqueous $NaHCO_3$ solution (50 mL) was added, and the residue was concentrated under reduced pressure to obtain a dry precipitate. Chloroform (100 mL) was added, and the mixture was stirred for 2h, then the solution was filtrate, washed with an excess of chloroform and concentrated under reduce pressure. The residue was purified by SiO_2 column chromatography using CH_2Cl_2/EtOH/NH_3 aq. (9/1/0.1) to obtain compound **<u>16</u>** (0.4 g, 79%) as a red solid.

ESI-MS= 338.2 $[M+H]^+$.

Synthesis of compound <u>18</u>.

To a stirred solution of compound **<u>16</u>** (0.40 g, 1.18 mmol) and **<u>17</u>** (0.25 g, 0.94 mmol) in CH_2Cl_2 (20 mL) was added AcOH to obtain a pH around 7. The mixture was stirred 4h at 25°C under argon atmosphere. After this time, $NaBH(OAc)_3$ (0.33 g, 1.55 mmol) was added and the reaction was continued overnight. At the end of reaction, a saturated aqueous $NaHCO_3$ solution (50 mL) was added, the mixture was extracted two times with DCM (2 x 100 mL), dried over Na_2SO_4, concentrated under reduced pressure and the residue was purified by SiO_2 column chromatography using DCM/EtOH (9/1) to give compound **<u>18</u>** (0.28 g, 50%) as a pink solid.

ESI-MS= 590.4 $[M+H]^+$.

Synthesis of compound 19.

To a stirred solution of compound **<u>18</u>** (85 mg, 0.14 mmol) in H_2O/EtOH (1mL/1.5mL), KOH (0.9 g) was added, and the mixture was stirred at 80°C overnight. After cooling, the solution was extracted two times with chloroform (2 x 30 mL), dried over Na_2SO_4, concentrated under reduced pressure and the residue was purified by reverse phase using H_2O/Acetonitrile (7/3) as eluant to obtain **TinyPol-NH** (32 mg, 45%) as a white solid.

HRMS-ESI: m/z: calcd for $C_{27}H_{48}N_5O_4^{2\cdot +}$ ($[M+H]^+$) 506.3701 found 506.3701.

Radical stability: Dinitroxides of the TinyPol series are stable radicals at room temperature in solution and in solid state. TinyPol solutions have been stored at -20°C and checked regularly by EPR (line shape and spin counting) during a 12-month period with no sign of degradation.

S2 NMR experiments

Sample preparation. The synthesis protocols of the dinitroxide radicals investigated here are reported above. AsymPol-POK was purchased from Cortecnet. The solvents used for sample formulation (d_8-glycerol and D_2O) were purchased from Sigma Aldrich. The solutions were prepared by dissolving ca. 1 mg of the radical powders in d_8-glycerol/D_2O/H_2O 60/30/10 (v/v/v). Depending on the biradical, solubilization up to the desired concentration was more or less straightforward. For TinyPol-NH, TinyPol(OH)$_4$, O-TinyPol(OH)$_4$, M- TinyPol and M-TinyPol(OH)$_4$ solutions, mechanical stirring at room temperature was sufficient to achieve a radical concentration of 10 mM. For the DNP measurements, ca. 2 µL of the solutions were pipetted into a 1.3 mm zirconia rotor that had been pre-packed with a small pellet of ground nail-polished KBr, in order to monitor the sample temperature by following ^{79}Br relaxation.

MAS DNP NMR experiments. All DNP experiments were performed on commercial Bruker Avance III wide bore spectrometers, operating at 18.8 T, and equipped with triple resonance 1.3 mm and 0.7 mm low-temperature MAS probes. DNP was achieved by irradiating the sample with high-power microwaves at frequencies of 527 GHz generated by a gyrotron that was operating continuously during the DNP experiments (stability of better than ±1% on the NMR signal). A microwave power of 22 W was used, measured via a calorimeter halfway through the waveguide. The main magnetic field position has been adjusted for optimal cross-effect and was found at the same position as AMUPol or TEKPol. One-dimensional proton NMR spectra were acquired with a DEPTH pulse sequence to suppress the background of the probes,[3] consisting in a π/2 pulse followed by two π pulses which are phase cycled according to a combined "EXORCYCLE" and "CYCLOPS" scheme. The duration of π/2 pulses was set to 2.5 µs, corresponding to a 100 kHz RF field. The spectra in presence and absence of microwave irradiation were taken using a recycle delay of $1.3 \times T_{B,ON}$. In all the experiments recorded in 1.3 mm rotors, the temperature of the microwave ON and OFF spectra was carefully monitored via ^{79}Br longitudinal relaxation and adjusted, if necessary, in order to ensure uniformity across the spinning frequency range and to compensate for sample heating in presence of microwave irradiation. Proton longitudinal relaxation times and DNP build-up times were measured with a standard saturation recovery sequence followed by a rotor synchronized echo period before signal acquisition. Zirconia rotors were used. While TinyPol was only soluble at 5 mM in d_8-glycerol/D_2O/H_2O 60/30/10 (v/v/v),[1]all the radicals studied in this work could be easily prepared at a concentration of 10 mM. The concentration dependence was tested for M-TinyPol(OH)$_4$ that highlighted a clear optimum at 10 mM, in line with results obtained previously on M-TinyPol[1] (the optimum radical concentration will depend on the deuteration level of the matrix[4]) .Under these experimental conditions, AMUPOL leads to an ε_H factor of 67 and a $T_{B,ON}$ of 12 s. The measurement of the depolarization factors as well as the calculation of Σ are detailed below.

S3 Continuous wave EPR

The experimental continuous wave (CW) EPR spectra of the dinitroxides have been acquired from a 200 μM water solution using a Bruker X-Band spectrometer (Table S1).

The fitting has been performed using the EasySpin package[5] using the "esfit" function with the pepper function with two or three components. The fitting procedure follows a so-called hybrid least-squares fittings ("hybrid method") where one optimization algorithm is used to locate a potential minimum, and another one is used to refine the parameters at that minimum. The first stage was performed using a genetic algorithm and the second one a Nelder/Mead simplex algorithm targeting in both cases the Fourier transform of the experimental and simulated spectra ("fft" option of the EasySpin function "esfit").

In Table S2 the mean isotropic value of the electronic g-factor, hyperfine coupling, and electron-electron *J*-coupling are reported. We note that all the spectra presented correspond to fast tumbling regime, considering the experimental temperature and concentration, thus all anisotropies (of g-tensor, hyperfine coupling and electron-electron *J*-coupling) are averaged out and their presence is only indicated by the differences in linewidths among different EPR lines in each spectrum. The overall quality of the fitting was estimated with the root-mean-square deviation (RMSD) of the experimental as simulated data without any further transformation. Note that the sign of the *J*-coupling cannot be determined from these solution EPR studies.

Since this approach does not allow for very accurate reproducing of the relative peak-to-peak line intensities, the corresponding accuracy was assumed to be rather poor, and the overall *J*-coupling fit parameters should be taken with cautions. In Figure S9 we report the comparison between the experimental data and the fits obtained with the procedure described above. In Figure S10 there are reported the individual components for each fit and the isotropic *J*-coupling values and relative weight.

Depending on the PAs, the best fits were found using two or three different isotropic *J* components and adding a gaussian line broadening of ca. 10-15 MHz for each component.

Compound<br>*MW (g.mol^{-1})*	Structure	solvent	EPR spectrum
TinyPol(OH)$_4$	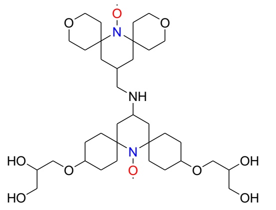	Water	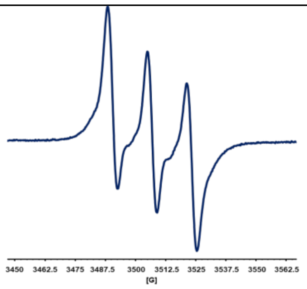
O-TinyPol	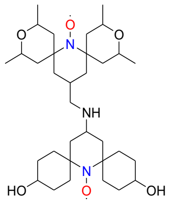	Water	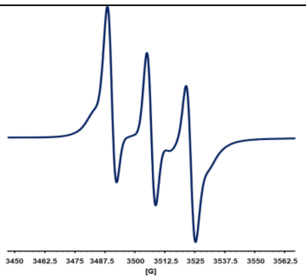
O-TinyPol(OH)$_4$	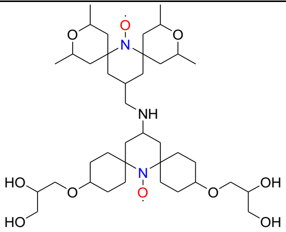	Water	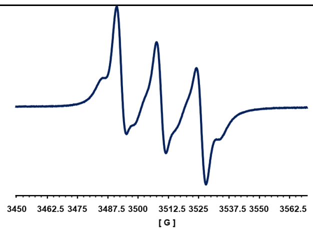
M-TinyPol(OH)$_4$	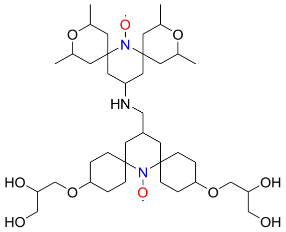	Water	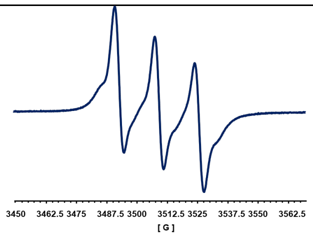
TinyPol-NH	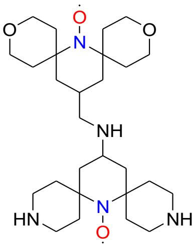	Water	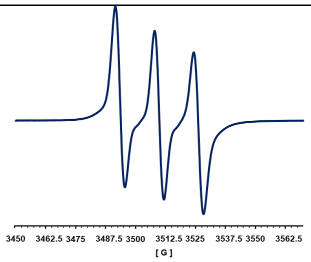
M-TinyPol(OH)$_4$-d$_{10}$	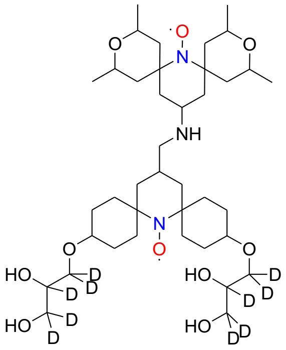	Water	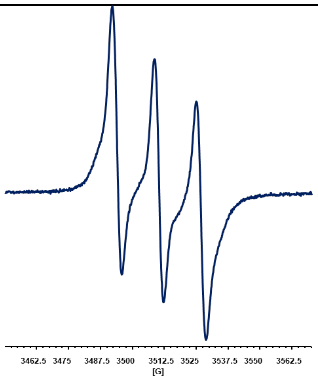

Table S1: Room temperature, 9 GHz EPR spectra of dinitroxide solution (0.2 mM).

Biradical	*g* iso	A iso / MHz	<*J*> / MHz	RMDS
TinyPol-NH	2.0058	46.3	14.1	$6.18 \cdot 10^{-2}$
O-TinyPol	2.0055	46.8	27.7	$8.10 \cdot 10^{-2}$
TinyPol(OH)$_4$	2.0058	46.7	29.2	$7.20 \cdot 10^{-2}$
O-TinyPol(OH)$_4$	2.0056	46.3	27.1	$6.10 \cdot 10^{-2}$
M-TinyPol	2.0055	47.1	25.3	$7.10 \cdot 10^{-2}$
M-TinyPol(OH)$_4$	2.0058	47.0	27.5	$6.97 \cdot 10^{-2}$

Table S2: Results from the fits of the EPR spectra for the TinyPol-like radicals investigated here. Except for TinyPol-NH, the results indicate a very similar distribution of *J*-couplings throughout the TinyPol series with average values between 25 and 35 MHz.

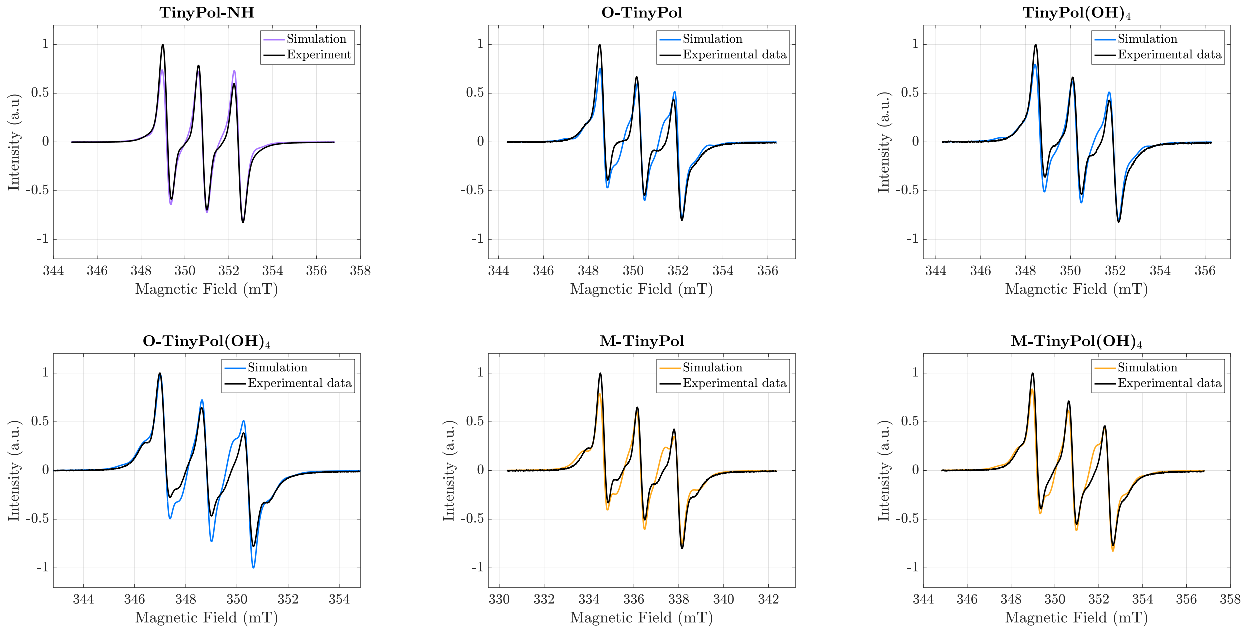

Figure S9: Experimental data (black) and fits (in color) of TinyPols X-band EPR spectra in water. The fitting procedure is detailed in section S2.

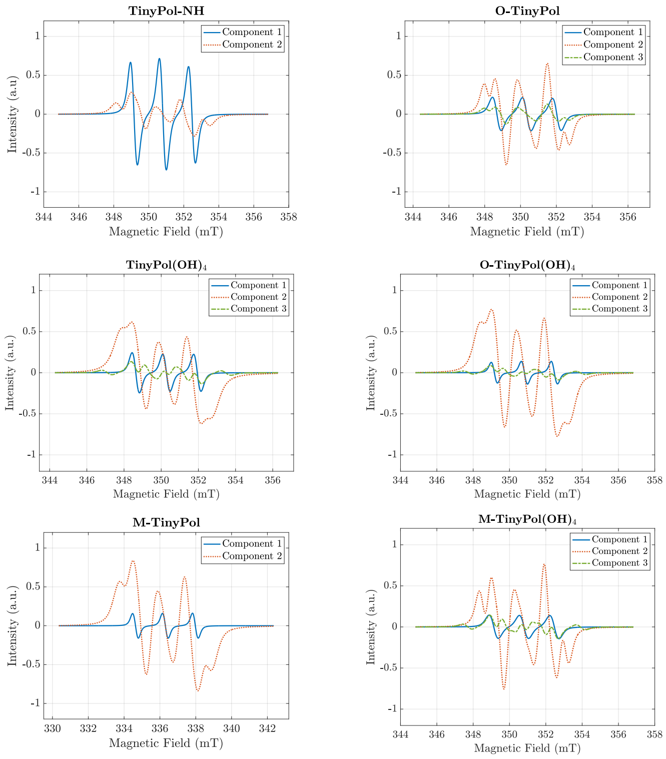

	J-coupling (MHz)	weight
TinyPol-NH	3.72<br>35.1	71.8 %<br>28.2 %
O-TinyPol	3.00<br>24.2<br>59.7	21.7 %<br>65.4 %<br>12.9 %
TinyPol(OH)$_4$	4.87<br>26.9<br>63.3	24.5 %<br>62.0 %<br>3.5 %
O-TinyPol(OH)$_4$	5.32<br>27.2<br>59.8	13.9 %<br>77.4 %<br>8.7 %
M-TinyPol	4.96<br>30.1	16.2 %<br>83.8 %
M-TinyPol(OH)$_4$	5.23<br>27.2<br>60.1	14.4 %<br>76.5 %<br>9.1 %

Figure S10: (Left) Weighed individual components of each fit. (Right) Isotropic *J*-coupling values and relative weight obtained by the EPR spectra of Figure S9.

S4 ESEEM EPR experiments.

For pulsed X- or W-band EPR experiments, roughly 60 µL of 100 or 200 µM solutions were placed into 3 mm outer diameter capillary tubes and inserted into the resonator after rapid freezing upon immersion in liquid nitrogen.

The deuterium ESEEM measurements were performed on a Bruker Elexsys E580 X-band EPR spectrometer equipped with a Bruker MS3 split-ring resonator at frequencies of approximately 9.3 GHz. The temperature of 50 K was achieved by liquid helium cooling. Three-pulse ESEEM measurements consist of the following sequence of pulses: π/2–τ–π/2-T-π/2–τ–echo.

The longitudinal electron magnetization is converted into transverse magnetization on allowed electron spin and nominally forbidden electron-nuclear spin transitions by the first 90° pulse. During the first delay τ, electron transverse magnetization dephases and the second 90° pulse flips most of the magnetization back to the z-axis. Part of the magnetization is converted to transverse magnetization on nuclear transitions. Longitudinal relaxation and transverse nuclear spin relaxation occur during time T and the final 90° pulse converts longitudinal electron spin magnetization and transverse nuclear magnetization into transverse magnetization on allowed and forbidden transitions. An echo signal is observed at time T+2τ. The value of τ = 344 ns was used to suppress proton modulations on the ESEEM decay envelope. The second inter-pulse delay, T, had an initial value of 80 ns and was incremented in steps of 8 ns. The integrated echo intensity was measured as a function of T increment, with an integration gate of 32 ns length. The pulse lengths were 16 ns for the 90° pulse and 32 ns for the π pulse in the echo. The intensity of the echo signal is therefore modulated by the hyperfine interaction between the electron spin and vicinal ^{2}H nuclei during the variable delay T, resulting in an oscillating decay.

In the three-pulse ESEEM experiment, the modulation depth, k_D, is defined as the peak-to-peak distance between the first maximum and the first minimum in the deuterium modulation. Fourier transformation of the normalized nuclear modulation function and computation of the absolute value provides a magnitude spectrum. The intensity of the peak at the ^{2}H frequency is proportional to the modulation depth k_D. The data were analyzed with a home-written MATLAB program. A 3rd-order polynomial background fitted to the primary data is subtracted from the primary data and the difference is divided by the background fit. The spectrum is obtained by apodization of the normalized nuclear modulation function with a Hamming window, zero filling the data to four times the original length, Fourier transformation and calculating the magnitude spectrum. The solvent accessibility parameter is defined as:

$$\Pi(\text{solvent}) = \frac{2k_D}{1-\cos(2\pi\nu_D\tau)} \times \left(\frac{\nu_D}{2MHz}\right) \quad (1)$$

It is independent of the measurement parameters due to compensation of the choice of inter-pulse delay τ and the exact static field B_0. Normalization to a standard value of 2 MHz allows for a direct comparison with data measured at different field. The deuterium modulation depth k_D is obtained by a least square fit to the normalized nuclear modulation function of a damped harmonic oscillation with fixed frequency ν_D, and variable damping constant and phase.

In Table S3 and S4 there are reported, respectively, the modulation depth and the solvent accessibility at 50 K parameter for 200 µM in DNP juice and the two different formulations reported in the main text (h_8-glycerol/D_2O, d_5-glycerol/H_2O both 60/40 v/v), while in Figure S11 and S12 there is reported the time dependent data and the correspondent magnitude spectra.

Radical	Modulation depth	Solvent accessibility
TinyPol-NH	0.19 ± 0.02	0.37 ± 0.04
O-TinyPol	0.20 ± 0.02	0.37 ± 0.04
TinyPol(OH)$_4$	0.17 ± 0.02	0.34 ± 0.03
O-TinyPol(OH)$_4$	0.18 ± 0.02	0.35 ± 0.03
M-TinyPol	0.18 ± 0.02	0.35 ± 0.03
M-TinyPol(OH)$_4$	0.17 ± 0.02	0.32 ± 0.03
AsymPol-POK	0.22 ± 0.02	0.43 ± 0.04

Table S3: ESEEM modulation depths and solvent accessibility parameters determined at X band for a series of 200 µM solutions of TinyPol-NH, O-TinyPol, TinyPol(OH)$_4$, O-TinyPol(OH)$_4$, M-TinyPol, M-TinyPol(OH)$_4$ and AsymPol-POK in d$_8$-glycerol/D_2O/H_2O 60/30/10 (v/v/v) (DNP juice) at 50 K.

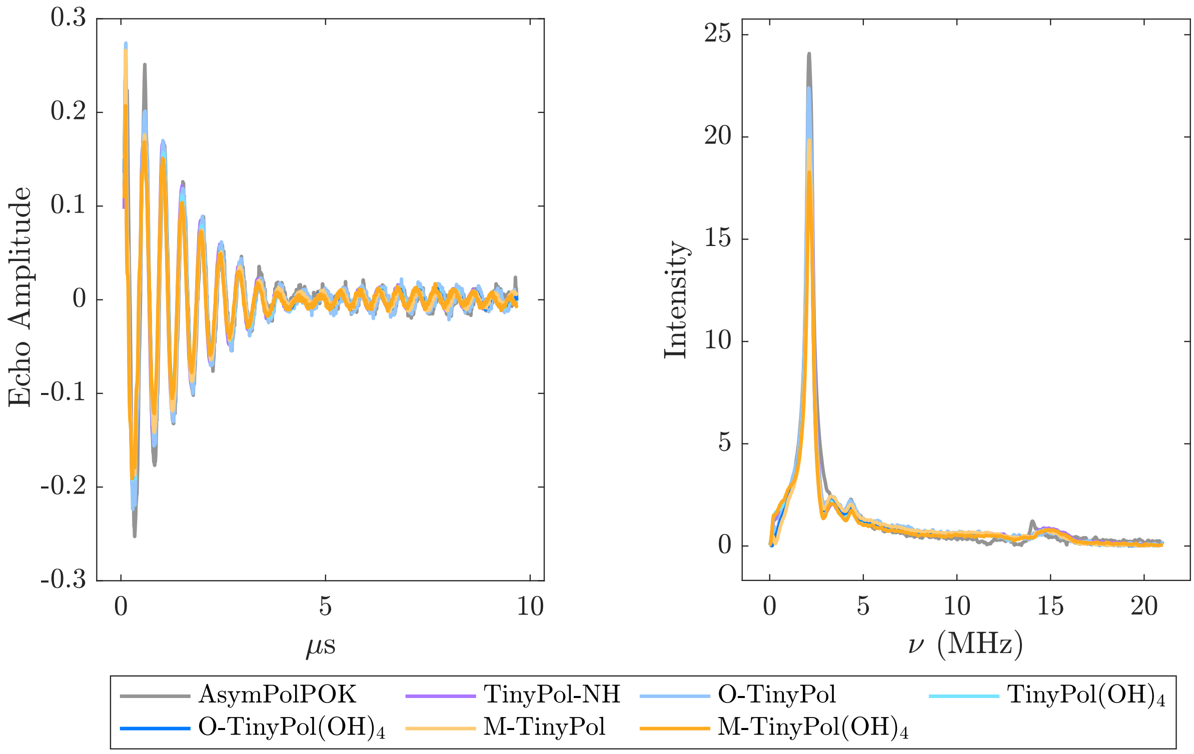

Figure S11: Three-pulse ESEEM time-domain data (left) and the corresponding magnitude spectra (right) for 200 µM solutions of O-TinyPol, M-TinyPol, TinyPol-NH, TinyPol(OH)$_4$, O-

TinyPol(OH)$_4$, M-TinyPol(OH)$_4$ and AsymPol-POK in d$_8$-glycerol/D_2O/H_2O 60/30/10 (v/v/v) (DNP juice) at 50 K.

Radical	Solvent 60/40 (v/v)	Modulation depth	Solvent accessibility
M-TinyPol	h$_8$-glycerol/D_2O	0.06 ± 0.01	0.10 ± 0.01
M-TinyPol	d$_5$-glycerol/H_2O	0.10 ± 0.01	0.19 ± 0.02
TinyPol(OH)$_4$	h$_8$-glycerol/D_2O	0.06 ± 0.01	0.10 ± 0.01
TinyPol(OH)$_4$	d$_5$-glycerol/H_2O	0.10 ± 0.01	0.20 ± 0.02
O-TinyPol	h$_8$-glycerol/D_2O	0.06 ± 0.01	0.11 ± 0.01
O-TinyPol	d$_5$-glycerol/H_2O	0.11 ± 0.01	0.23 ± 0.02

Table S4: X-band ESEEM modulation depths and solvent accessibility parameters for 200 µM solutions of M-TinyPol, TinyPol(OH)$_4$ and O-TinyPol radicals in different glycerol/water mixtures at 50 K.

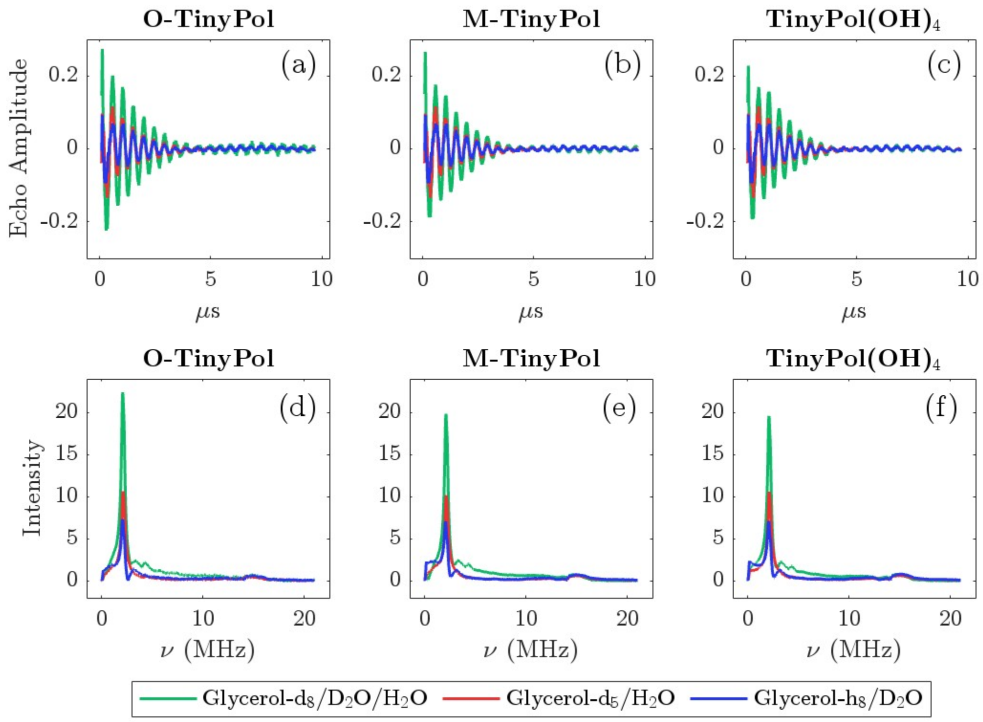

Figure S12: Three-pulse ESEEM time-domain data and the corresponding magnitude spectra for 200 µM solutions of M-TinyPol, TinyPol(OH)$_4$ and O-TinyPol radicals in different glycerol/water mixtures at 50 K.

S5 Electron spin T_{ir} and T_m measurements

Low-temperature W-band EPR measurements were carried out on a Bruker E680 spectrometer at a microwave frequency of 94 GHz and a temperature of 105 K, on 100 μM frozen solutions of the dinitroxide radicals in d_8-glycerol/D_2O/H_2O (60/30/10 v/v). π/2 and π pulses of 52 and 104 ns were used, respectively. Relaxation measurement was carried out at the maximum intensity of the EPR spectra, recorded using field-dependent echo-detection (ED EPR). Longitudinal relaxation (T_{ir}) times were recorded with the inversion recovery sequence, and transverse relaxation (T_m) times were recorded using a variable delay Hahn echo sequence, with a phase cycle on the π/2 pulse. The Hahn echo delay time for the inversion recovery detection and the initial echo delay in the transverse relaxation measurements was set to 400 ns. The data were fit with a single stretched exponential to account for a distribution of the values. A similar analysis was recently done for hybrid trityl-nitroxide radicals.[6] The first moment of this distribution is indicated as the mean value (as described below). All the fittings were performed using home-written MATLAB scripts and reported in Figure S13 and S14.

In analogy to Zagdoun *et al.*[7] and Lund *et al.*[1], longitudinal electron spin relaxation time traces were fitted using a stretched exponential function:

$$I(t) = I_0 + I_1 \cdot \mathrm{e}^{-\left(\frac{t}{T_{1e}^*}\right)^{\beta}} \qquad (2)$$

where I_0 is the initial intensity, I_1 the proportionality factor, T_{1e}^* the decay time parameter and β the stretching parameter. The mean relaxation time is given as the first moment $\langle T_{1e} \rangle$ of the distribution as a mean, called the inversion recovery time T_{1r}:

$$T_{1r} = \langle T_{1e} \rangle = \int_0^{\infty} \mathrm{e}^{-\left(\frac{t}{T_{1e}^*}\right)^{\beta}} dt = \frac{T_{1e}^*}{\beta}\, \Gamma\left(\frac{1}{\beta}\right) \qquad (3)$$

Where Γ is the Gamma function.

The transverse electron spin relaxation times were recorded using a variable delay Hahn echo sequence and fitted with a stretched exponential.

$$I(t) = I_0 \cdot \mathrm{e}^{-\left(\frac{t}{T_{2e}^*}\right)^{\beta}} \qquad (4)$$

where I_0 is the initial intensity, T_{2e}^* the decay time parameter and β the stretching parameter. The mean relaxation time is given as the first moment $\langle T_{2e} \rangle$ of the distribution as a mean, called the phase memory time T_m:

$$T_m = \langle T_{2e} \rangle = \int_0^{\infty} \mathrm{e}^{-\left(\frac{t}{T_{2e}^*}\right)^{\beta}} dt = \frac{T_{2e}^*}{\beta}\, \Gamma\left(\frac{1}{\beta}\right) \qquad (5)$$

Where Γ is the Gamma function.

Biradical	T_{1r} (μs)	β
TinyPol-NH	306	0.81
O-TinyPol	284	0.75
TinyPol(OH)$_4$	250	0.81
O-TinyPol(OH)$_4$	258	0.79
M-TinyPol	256	0.78
M-TinyPol(OH)$_4$	251	0.80
AsymPol-POK	199	0.75

Table S5: T_{1r} and β values for the TinyPol-like radicals resulted from the fitting of the experimental data using a stretched mono-exponential.

Biradical	T_m (μs)	β
TinyPol-NH	6.8 ± 0.4	1.13
O-TinyPol	3.3 ± 0.4	0.62
TinyPol(OH)$_4$	6.9 ± 0.4	1.14
O-TinyPol(OH)$_4$	2.8 ± 0.1	0.65
M-TinyPol	1.9 ± 0.2	0.53
M-TinyPol(OH)$_4$	2.9 ± 0.1	0.68
AsymPol-POK	<2.00	nd

Table S6: T_m and β values for the TinyPol-like radicals resulted from the fitting of the experimental data using a stretched mono-exponential.

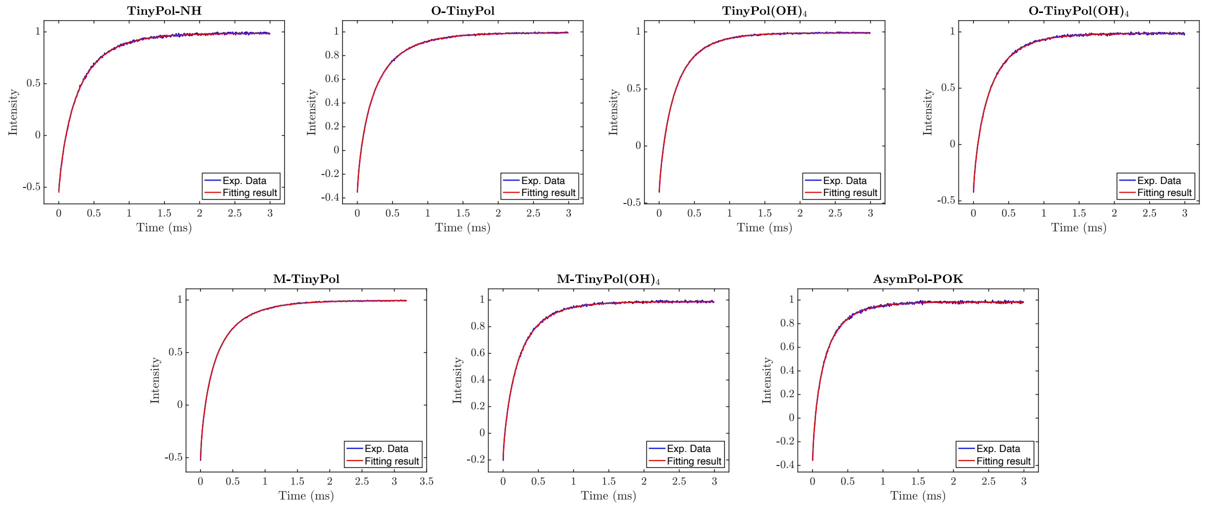

Figure S13: Fitting results of the experimental electron spin T_1 relaxation times at W band, at 105 K.

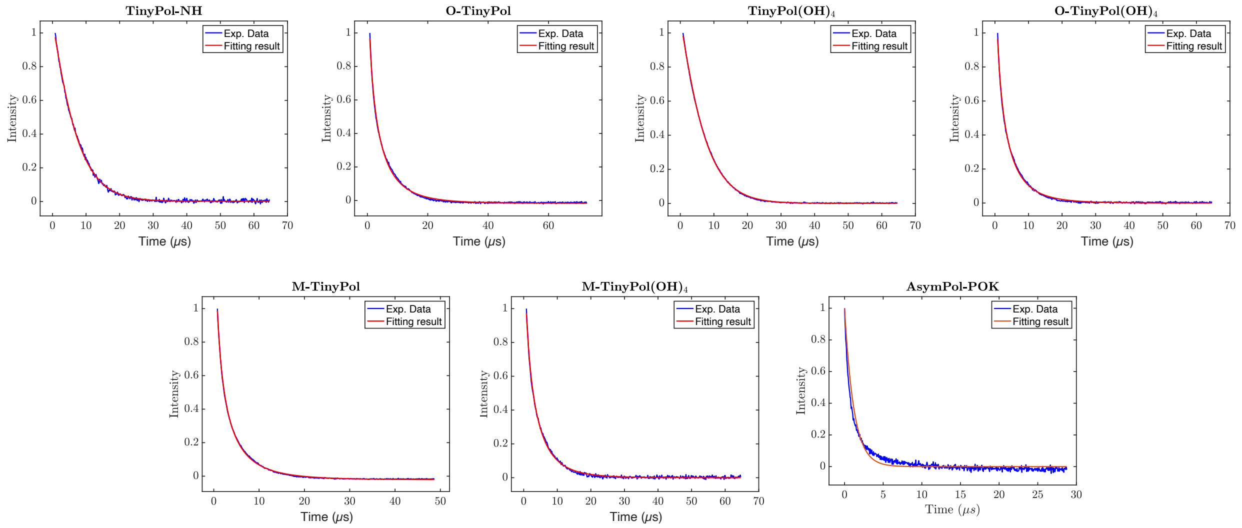

Figure S14: Fitting results of the electron spin T_2 relaxation times at W band, at 105 K.

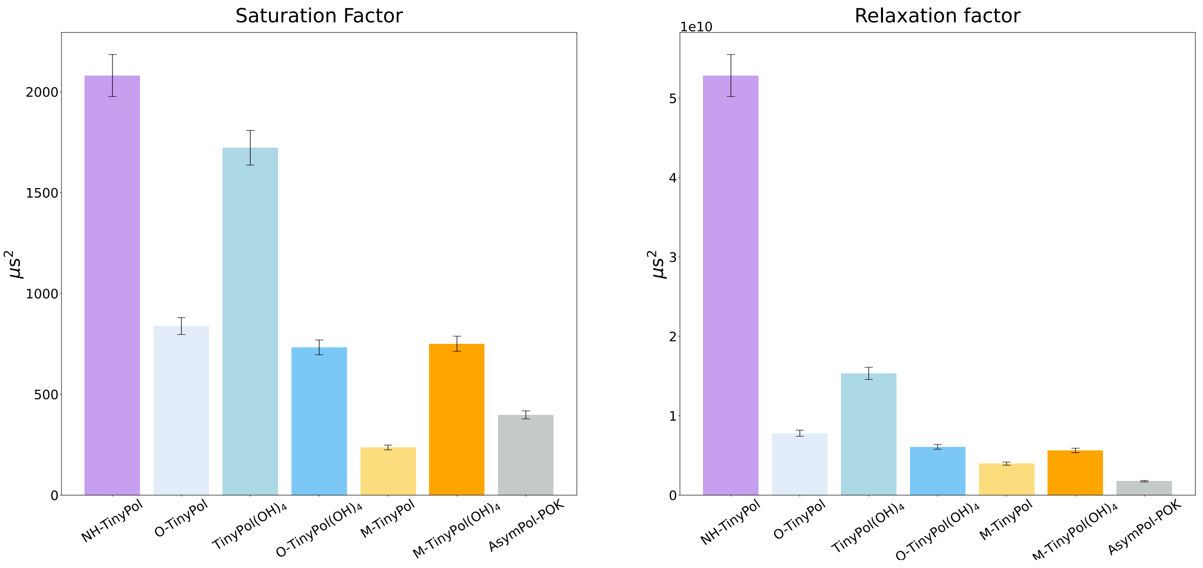

Figure S15: (a) Saturation factor and (b) relaxation factors for the radicals investigated here. The saturation factor is defined as the product of T_{ir} and T_m while the relaxation factor is the product of $T_{ir,}$, T_m and the proton polarization build-up time measured with microwave on irradiation, $T_{B,ON}$.[8] For AsymPol-POK T_m is assumed to be 2.00 μs.

S6 Depolarization factor and overall sensitivity gain

The depolarization factor was measured from 1D ^1H spectra by taking the ratio the NMR signal intensity (per unit of mass) of the frozen solution with radical divided by the NMR signal intensity (per unit of mass) of the same solution in the static regime, both measured with a recycle delay of 5*T_1. The factor was then calculated according to the following equation:

$$\varepsilon_{DEPO} = \frac{I_{with\ radical,\mu w\ off\ (\omega_r)}}{I_{with\ radical,\mu w\ off\ (static)}} \qquad (6)$$

These measurements were done at different spinning frequency. This factor does not take into account the paramagnetic bleaching induced by the radical, but includes depolarization losses.

The overall sensitivity gain Σ' was calculated according to equation 7:

$$\Sigma' = \varepsilon \cdot \varepsilon_{DEPO} \cdot \sqrt{\frac{T_{1,solvent}}{T_{B,on}}} \qquad (7)$$

where ε is the enhancement factor calculated as the ratio of signal intensity microwave on divided by signal intensity microwave off, ε_{DEPO} is the depolarization factor defined in equation (6), $T_{1,solvent}$ and $T_{B,on}$ are the build-up times of respectively the undoped DNP matrix and the DNP matrix containing the radical in presence of microwave irradiation. Here a value of 143 s was measured for the undoped DNP matrix at 40 kHz MAS and was considered for the calculations.

O-TinyPol(OH)$_4$			
MAS (kHz)	^1H Enhancement	$T_{b,ON}$ (s)	Depolarization
40	159	8.3	0.68
M-TinyPol			
MAS (kHz)	^1H Enhancement	$T_{b,ON}$ (s)	Depolarization
40	124	18.8	0.86
20	141	14.9	0.84
10	138	14.7	0.83
M-TinyPol(OH)$_4$			
MAS (kHz)	^1H Enhancement	$T_{b,ON}$ (s)	Depolarization
40	138	7.50	0.68
30	142	7.25	0.75
20	153	6.27	0.85
10	161	6.12	0.80
AsymPol-POK			
MAS (kHz)	^1H Enhancement	$T_{b,ON}$ (s)	Depolarization
40	75.0	4.36	0.66
30	60.0	3.27	0.72
20	62.0	2.95	0.78
10	58.0	2.76	0.86

Table S7: Values used to calculate the Σ' factor.

S7 DFT geometry optimization

DFT geometry optimization of the TinyPol-like radical structures has been performed using ORCA 5[9] at a B3LYP level with a TZVP basis set. The optimized geometries are reported in Figure S16 The input structure has been generated and pre-optimized using Avogadro.[10]

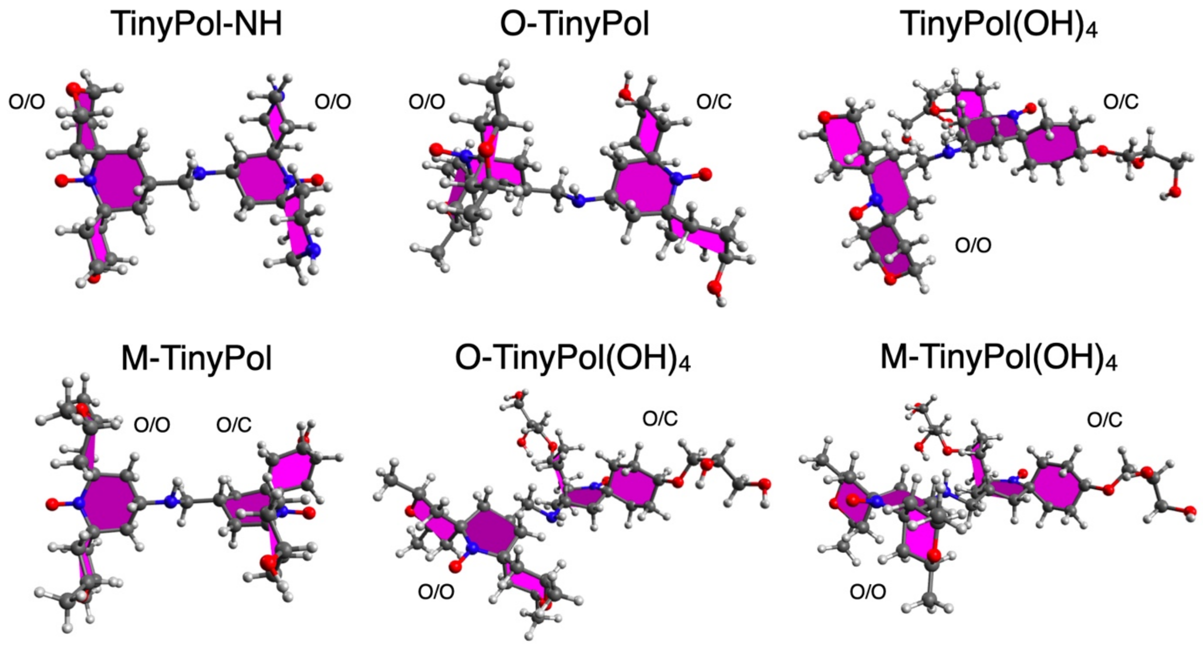

Figure S16: DFT-optimized geometries of the new TinyPols derivatives. The open-closed conformations are indicated with O/O (open/open) and O/C (open/closed).

S8 Molecular Dynamics Simulations

Molecular Dynamics simulation have been performed in NPT ensemble carried out at 293 K in a mixed solvent composed by water (TIP3P) and glycerol (40/60 v/v) to mimic the DNP juice, with the GROMCAS 2019 package.

The topologies for the different dinitroxides have been constructed in the following way:
Different structures have been optimized (HF/SVP) and after the atomic charges have been determined by restricted electrostatic potential (RESP) fitting using GAUSSIAN 09.[11] The .mol2 files have been generated from the RESP charge calculation using the antechamber module of the AmberTools package. At this stage, the lone pair on the nitroxide oxygen atom has been included following the literature approach.[12] Using the tleap module of AmberTools the bi-nitroxide has been put into a cubic box filled with water molecules.

The final GROMACS topology file has been generated with the antechamber python parser interface (acpype) module for the construction of initial topology files. After an energy minimization of the molecule-water system, the glycerol molecules have been added up to the target concentration using the GROMACS "insert-molecules" command, finalizing the .gro file for the simulation.

It has been used the AMBER force field (FF89SB) which includes parameters optimized by Barone et al. for the N-O• group.[12] For each simulation the molecule was embedded in a cubic box containing about 1100 solvent molecules. After an energy minimization of the whole system, the temperature and the pressure were equilibrated by performing respectively NVT and NPT simulation over 500 ps (293 K, 1 atm). The production runs of the simulations have been performed in the NPT ensemble up to a duration of 100 ns. A total of 5 different runs has been performed to probe the conformational space providing different starting velocity.

Our simulation setup implicitly disregards any possible interaction among radicals, because a single dinitroxide molecule is present in the simulation box which dimensions are also enough to prevent the molecule to interact with its mirror images generated by the periodic boundary conditions.

In Figure S17 and S18, we report the cumulative distribution of the nitroxides electron-electron distance and of the normal vector of the mean planes (defined as Figure S19) of the nitroxide moieties considering 5 different 100 ns run for each biradical. The electron-electron distance has been calculated considering the mean point of the N and O atoms of the NO group.

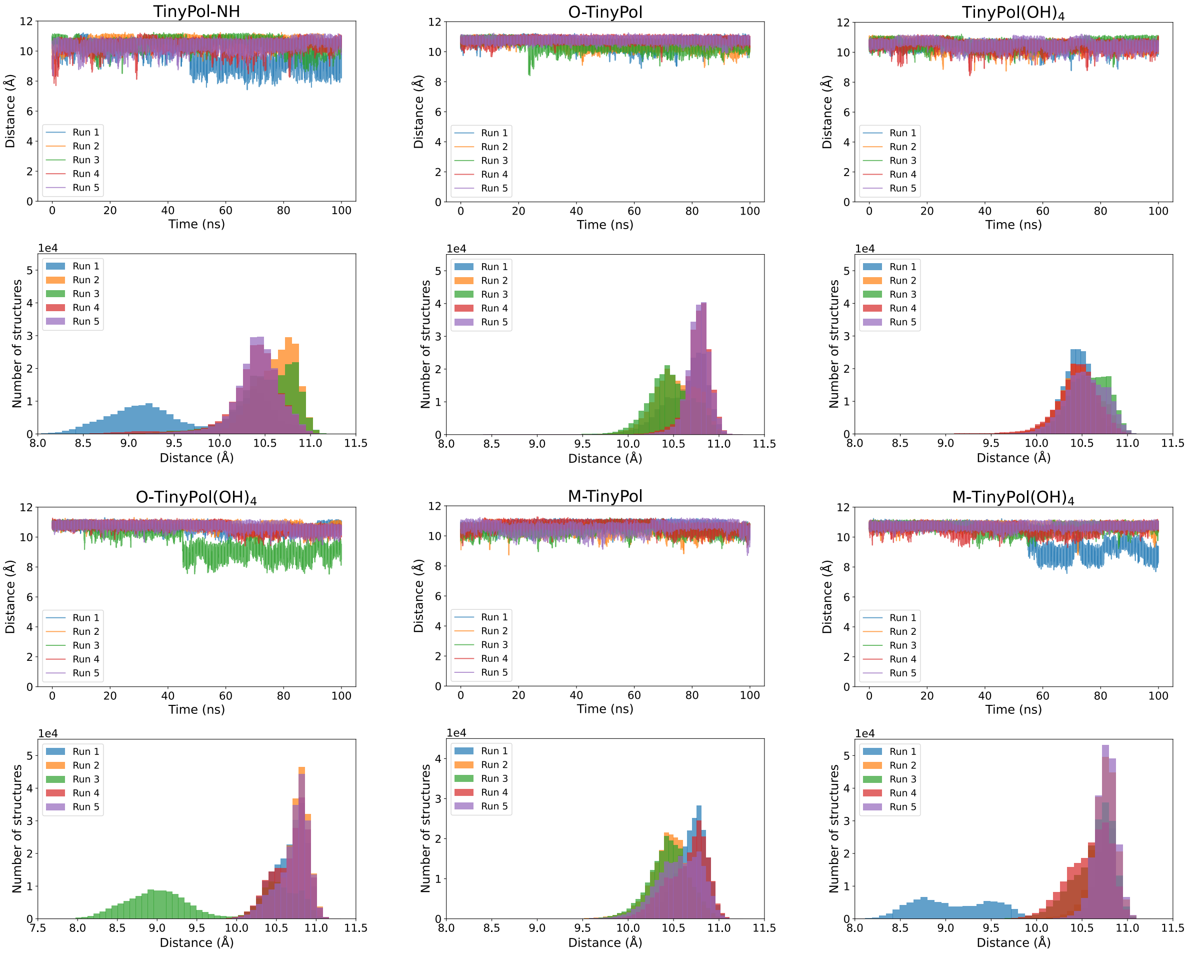

Figure S17: Electron-electron distance distribution for each TinyPol derivative. Each plot shows the result of 5 different runs.

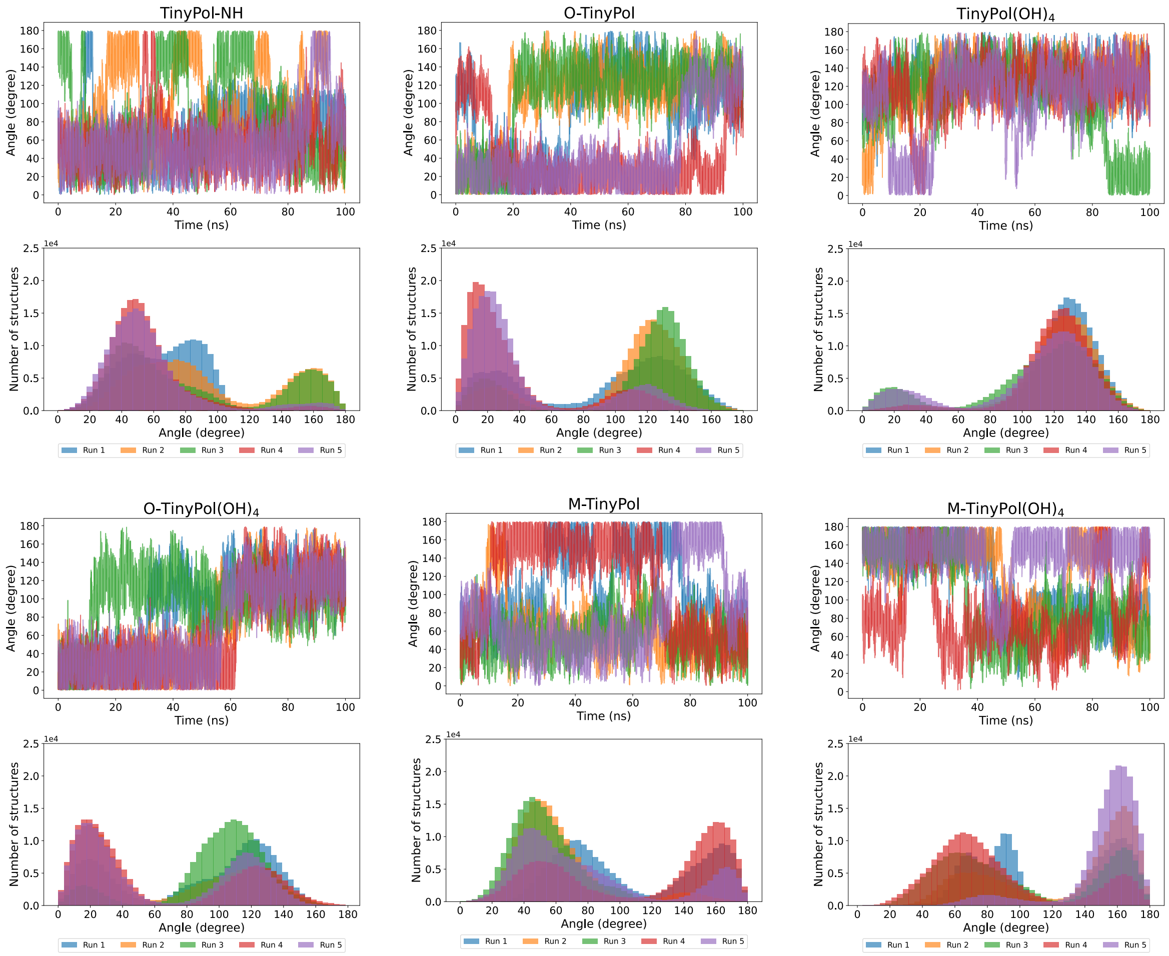

Figure S18: Mean plane angle distribution for each TinyPol derivative. Each plot shows the result of 5 different runs.

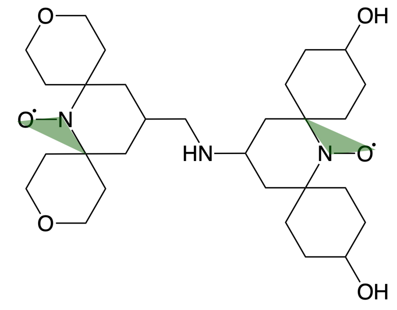

Figure S19: Planes used to define the orientation of the two g-tensors.

S9 Proton density distribution around radical

To obtain the proton densities around the radicals, the number of protons at a distance $[d, d + \Delta]$ from each radical (assumed to be localized in the middle of the N—O bond) was extracted and divided by the volume of the corresponding spherical slice selected. We chose Δd = 0.1 Å. The densities around the two radicals in each compound were averaged to yield the final proton densities.

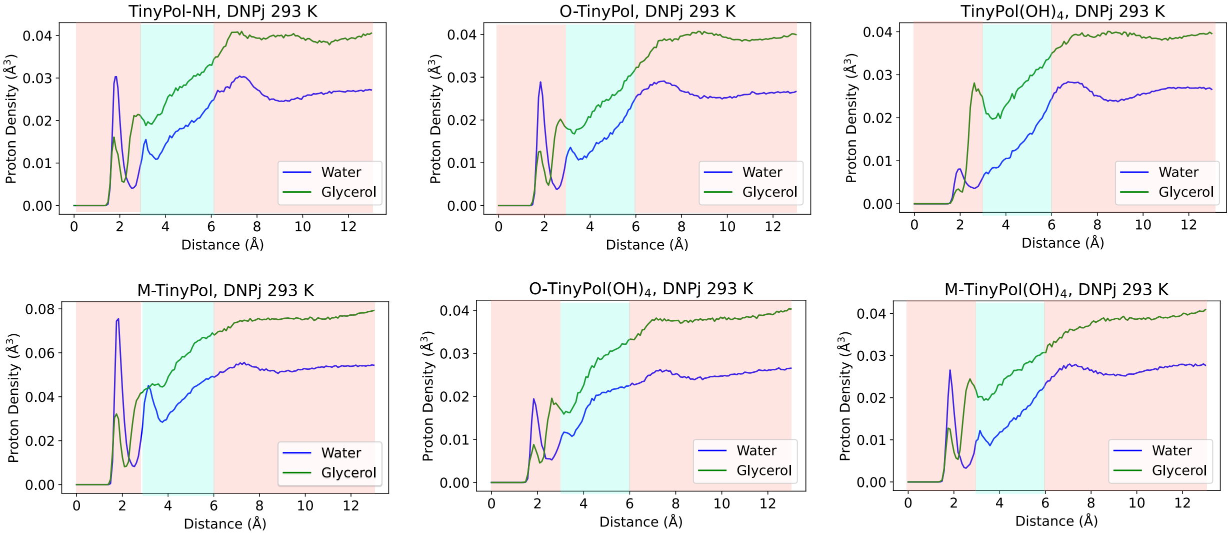

Figure S20: Radial ^{1}H distribution for the TinyPol-NH, O-TinyPol, TinyPol(OH)$_4$, O-TinyPol(OH)$_4$, M-TinyPol, M-TinyPol(OH)$_4$, in a mixed solvent (water and glycerol).

S10 DNP performance in degassed solutions

The experimental values reported in Table 1 were measured without degassing the solutions.
However, it is well known that the paramagnetic impurities such as paramagnetic oxygen dissolved in polarizing solutions can have a significant impact on the overall DNP performances. This is due to their role in hindering the spin-diffusion process, as explained by the "source-sink" model.
Under microwave irradiation, in a bulk solution, the polarization is uniformly distributed from the electron sources (the polarizing agents) by ^{1}H-^{1}H spin-diffusion. The presence of paramagnetic impurities in the solution that will act as "relaxation sinks", accelerating relaxation toward thermal equilibrium. In 2017 we showed that such relaxation sinks, even present at small concentrations (of the order of μM or tens of μM), modulate the overall efficiency of the DNP process, and explain the increase of ε_H and $T_{B,ON}$ as a function of MAS frequency.[13] There, the source-sink diffusion model for polarization transfer was implemented to interpret experimental Overhauser (OE) DNP data recorded with 1,3-bisdiphenylene-2-phenylallyl (BDPA) dissolved in o-terphenyl (OTP).
Oxygen dissolved in the bulk solutions is expected to be the main source of paramagnetic sink, especially in non protic organic solvents, such as tetrachloroethane (TCE). We have recently shown that a sink concentration of ~50 mM could explain the MAS dependence of ε_H for HyTEK2 in TCE solutions.[14] DNP formulations using organic solvents are typically degassed by a series of rotor insertion and ejection.[15]
Extensive testing of water-soluble radicals in aqueous solutions are usually performed in non-degassed solutions. This is because the oxygen solubility in water is much lower than in organic solvents.[16] The O_2 solubility in mixtures of water and glycerol is even lower than that in pure water.[17]
Nevertheless, it was recently observed by Griffin and co-workers that the use of carefully degassed water-glycerol mixtures has an significant impact on the OE enhancement of BDPA in ortho-terphenyl (OTP) and of NMe_3-BDPA in DNP juice, with an improvement of ~ almost a factor 2.[18] Compared to CE dinitroxides, BDPA derivatives for OE DNP lead to much longer polarization build-up times.[19] The effect of paramagnetic O_2 in the solution is therefore expected to be weaker for dinitroxides (Figure S22).
In order to evaluate this effect, measurements were carried out in 10 mM degassed solution of O-TinyPol$(OH)_4$. A degassed water-glycerol solution (60% d_8-glycerol, 30% D_2O and 10% H_2O, (v/v/v)) was prepared in the glove box from D_2O, H_2O and glycerol-d_8 solutions that were carefully degassed separately. Degassing was achieved by bubbling nitrogen gas through the solvents for an appropriate amount of time (several hours) to displace dissolved air and oxygen, using a Schlenk line system.
The rotor has then been packed inside the glove box and quickly transferred to the NMR probe in a protected atmosphere. Then the rotor was quickly inserted inside the LTMAS DNP probe cooled with ultra-pure N_2 gas. For the entire duration of the sample preparation, O_2 level inside the globe box was maintained below 0.5 ppm.
Both the enhancement factors and build-up times of the degassed polarizing solution were measured and compared with the same solution that was kept open over the bench overnight.
The results show only a moderate improvement in the enhancement factors ε_H (an average improvement of 15 % over the values measured at three different spinning frequencies) and a marginal improvement in the polarization build-up times $T_{B,ON}$ (an average improvement of 8 % over the values measured at three different spinning frequencies) using a degassed solution. This results in an average sensitivity increase of 11%.

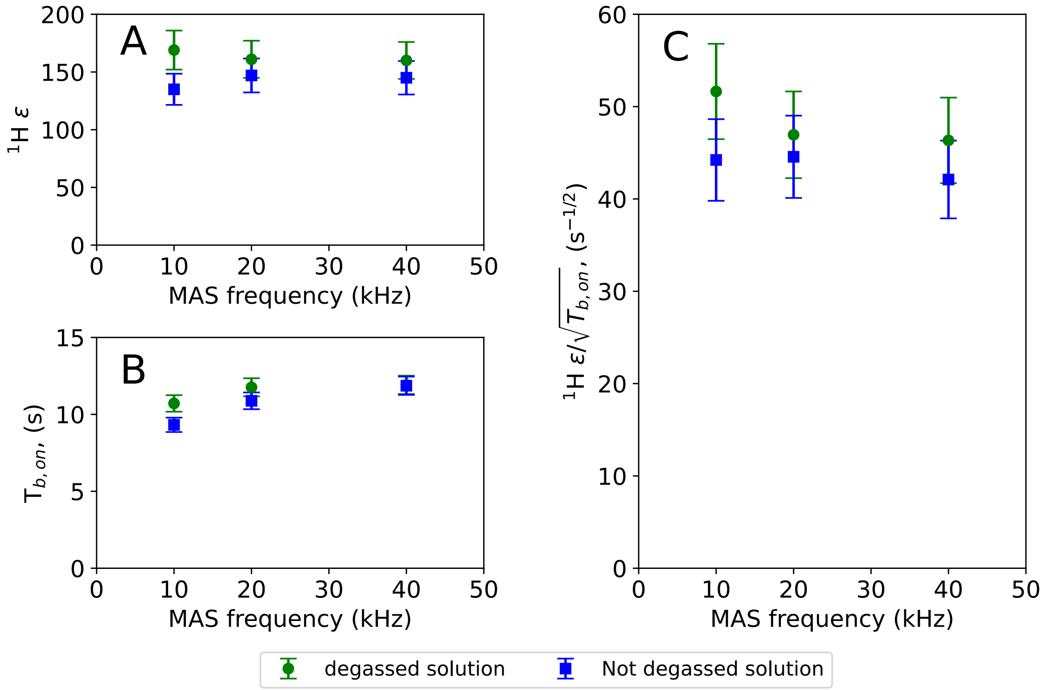

Figure S21: ^{1}H enhancement factors (A) and $T_{B,on}$ (B) measured via ^{1}H-^{13}C CP experiments, together with calculated sensitivity factors (C) for degassed and 10 mM O-TinyPol(OH)$_4$ in degassed and non-degassed solutions (60% d_8-glycerol, 30% D_2O and 10% H_2O, (v/v/v)). The measurements were performed at 18.8 T (800 MHz) at a temperature of 105±5 K.

The role played by oxygen in reducing the hyperpolarization can be well explained by the source-sink model.[13] In this model the dependence of the polarization (and consequently of the DNP enhancement) is explained through the presence of a polarization source (i.e. the polarization agent) and of a tiny amount of paramagnetic relaxing agents, the so-called "polarization sink" which is represented here by oxygen paramagnetic impurities. As explained in the original publication, paramagnetic impurities even of the order of μM are sufficient to act as polarization sinks.

The hyperpolarization that is uniformly distributed in the sample by the high concentration of sources is reduced by the action of polarization sink. The spin-diffusion process, described by the steady-state Fick diffusion equation:

$$D \cdot \left(\frac{\partial^2 P(r,t)}{\partial r^2} + \frac{2}{r}\frac{\partial P(r,t)}{\partial r}\right) - \frac{P(r,t) - P_0}{T_B} = 0 \qquad (6)$$

propagate the hyperpolarization toward the sinks that rapidly bring them to thermal equilibrium. In equation (6), D is the diffusion constant, P is the polarization and P_0 is the equilibrium value of the polarization, r is the radial distance from the sink and T_B is the polarization build-up time. The faster the spin diffusion or the higher is the oxygen concentration, the more rapidly is the hyperpolarization destroyed by the sink and the overall polarization gain reduced.

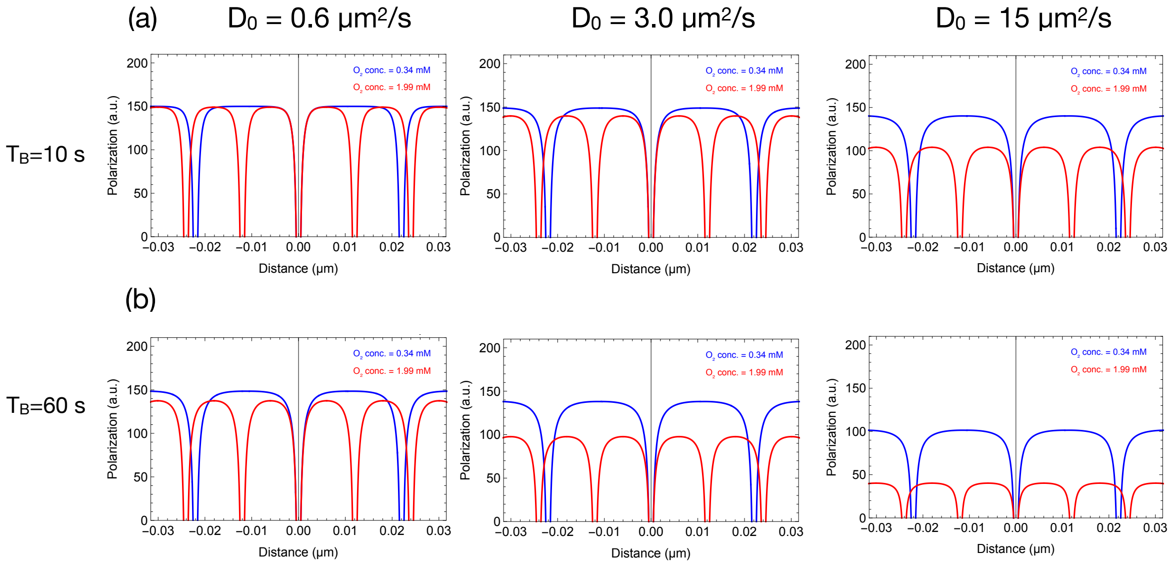

Figure S22: Simulations showing the radical polarization distribution in the sample as a function of oxygen concentration for 0.34 mM of O_2 (blue line) and 1.99 mM (red line) and for three different proton spin-diffusion rates. Simulations are done for a polarization build-up time of 10 s (a) and 60 s (b) The horizontal axis corresponds to the distance between the relaxation sinks (O_2 molecules here). The figure illustrates that degassing the DNP matrix is more crucial when the polarizing solution has an intrinsic long build-up time.

In Figure S22 we report the simulated profile of the steady-state hyperpolarization in the presence of different concentrations of sinks. In the simulation, the thermal polarization is imposed a value of 1 in the y-axes and the maximum hyperpolarization (in the absence of oxygen) is set to a value 150. Oxygen molecules are assumed to be equally spaced at the average distance expected for a concentration of 0.34 mM (blue line) and 1.99 mM (red line). Approaching the oxygen molecule, the polarization is reduced and drops to 1 at the R_0 radius of 5 Å from the molecule

In Figure S22 (a), is it possible to see how, increasing the spin diffusion rate D, the polarization is more rapidly delivered to the sinks and the overall polarization decreases. Also, when the sink concentration is increased (red line) the hyperpolarization is reduced.

In the panels (b), the bulk polarization build-up time T_B in the absence of sinks was increased from 10 s to 60 s. If the build-up time is slower, the polarization has more time to diffuse toward the sinks, and the impact of the oxygen concentration is stronger.

Simulations were performed on the basis of the of the spherical radial polarization distribution described in the equation 13 in the Supporting Information of ref.[13], with a value of $P_0 = 150$, $P_{sink} = 1$, $R_0 = 5.0$ Å, and the sink-to-sink average distance 2L calculated on the basis of the O_2 concentration of 0.34 mM (L = 10.5 nm) and 1.99 mM (L = 6.02 nm). The spin diffusion rate was calculated for a MAS frequency of 40 kHz obtained from equation:

$$D = \frac{D_0}{1 + k\ v_r} \quad (7)$$

where D_0 is the static spin diffusion constant, v_r is the MAS frequency in Hz and k is set to k= 6.25 10^{-3} s.

S10 Finite element simulations

(a)

(b)

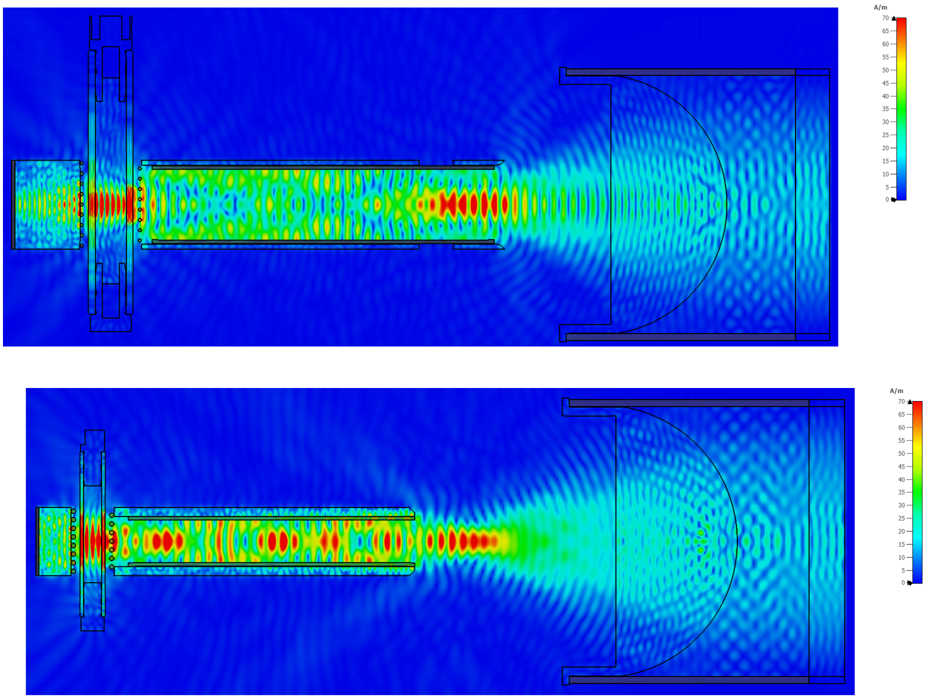

Figure S23: Microwave field penetration inside the rotor determined by finite element simulations. Calculations were done for a microwave frequency of 527 GHz in (a) for a 1.3 mm probe of optimized design[20] and in (b) for a 0.7 mm probe. For (a) the mean B_1 field in the active sample volume is ~38.1 µT/W$^{-1/2}$, which corresponds to ~1.07 MHz/W$^{-1/2}$. For (b) the mean B_1 is ~63 µT/W$^{-1/2}$, which corresponds to ~1.76 MHz/W$^{-1/2}$. Due to the geometric arrangement of the rotor**,** the bearings and the coil, the sample volume enlightened by the beam is about 10% larger compared in the 0.7 mm rotor. Drawbacks of the 0.7 mm setup are the less efficient coupling of the beam to the smaller diameter and the smaller radius of the zirconia wall, which can cause more scattering when the beam hits the rotor. The numerical field simulations were conducted using CST Microwave Studio 2017 (CST, Darmstadt, Germany).

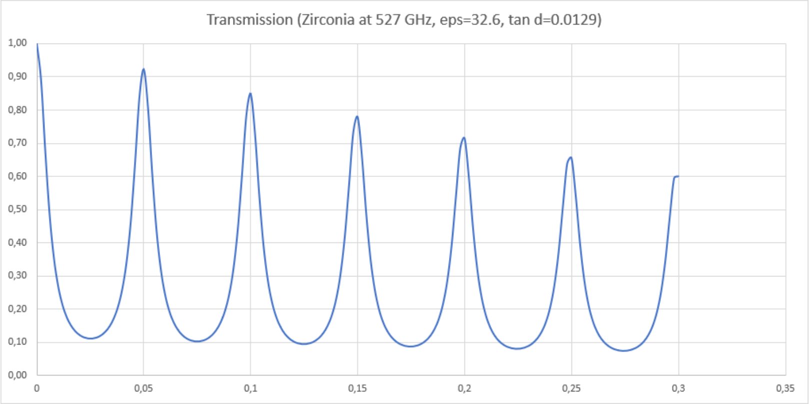

Figure S24: Adsorption of the microwave field as a function of the wall thickness (in mm). The periodic behavior reflects constructive and destructive interferences between the wall thickness and the wavelength. For 0.7 mm as well as 1.3 mm rotors (wall thickness of 0.1 and 0.2 mm respectively), the transmission is ideal. However, larger wall thickness leads to more

loss. This corresponds to 85% transmission in a 0.1mm slab as opposed to 71% transmission in a 0.2 mm slab.

S11 References

[1] A. Lund, G. Casano, G. Menzildjian, M. Kaushik, G. Stevanato, M. Yulikov, R. Jabbour, D. Wisser, M. Renom-Carrasco, C. Thieuleux, F. Bernada, H. Karoui, D. Siri, M. Rosay, I. V. Sergeyev, D. Gajan, M. Lelli, L. Emsley, O. Ouari, A. Lesage, *Chem. Sci.* **2020**, *11*, 2810-2818.

[2] W. M. Yau, K. R. Thurber, R. Tycko, *J. Magn. Reson.* **2014**, *244*, 98-106.

[3] M. R. Bendall, R. E. Gordon, *J. Magn. Reson.* **1983**, *53*, 365-385.

[4] N. A. Prisco, A. C. Pinon, L. Emsley, B. F. Chmelka, *Phys. Chem. Chem. Phys.* **2021**, *23*, 1006-1020.

[5] S. Stoll, A. Schweiger, *J. Magn. Reson.* **2006**, *178*, 42-55.

[6] A. M. Carroll, S. Eaton, G. Eaton, K. W. Zilm, *J. Magn. Reson.* **2021**, *322*.

[7] A. Zagdoun, G. Casano, O. Ouari, M. Schwarzwälder, A. J. Rossini, F. Aussenac, M. Yulikov, G. Jeschke, C. Copéret, A. Lesage, P. Tordo, L. Emsley, *J. Am. Chem. Soc.* **2013**, *135*, 12790-12797.

[8] D. J. Kubicki, G. Casano, M. Schwarzwälder, S. Abel, C. Sauvée, K. Ganesan, M. Yulikov, A. J. Rossini, G. Jeschke, C. Copéret, A. Lesage, P. Tordo, O. Ouari, L. Emsley, *Chem. Sci.* **2016**, *7*, 550-558.

[9] F. Neese, *Wiley Interdisciplinary Reviews-Computational Molecular Science* **2022**, *12*.

[10] M. D. Hanwell, D. E. Curtis, D. C. Lonie, T. Vandermeersch, E. Zurek, G. R. Hutchison, *Journal of Cheminformatics* **2012**, *4*.

[11] M. J. Frisch, G. W. Trucks, H. B. Schlegel, G. E. Scuseria, M. A. Robb, J. R. Cheeseman, G. Scalmani, V. Barone, B. Mennucci, G. A. Petersson, H. Nakatsuji, M. Caricato, X. Li, H. P. Hratchian, A. F. Izmaylov, J. Bloino, G. Zheng, L. Sonnenberg, M. Hada, M. Ehara, K. Toyota, R. Fukuda, J. Hasegawa, M. Ishida, T. Nakajima, Y. Honda, O. Kitao, H. Nakai, T. Vreven, J. A. Montgomery, J. E. Peralta, F. Ogliaro, M. Bearpark, J. J. Heyd, E. Brothers, K. N. Kudin, V. N. Staroverov, R. Kobayashi, J. Normand, K. Raghavachari, A. Rendell, J. C. Burant, S. S. Iyengar, J. Tomasi, M. Cossi, N. Rega, J. M. Millam, M. Klene, J. E. Knox, J. B. Cross, V. Bakken, C. Adamo, J. Jaramillo, R. Gomperts, R. E. Stratmann, O. Yazyev, A. J. Austin, R. Cammi, C. Pomelli, J. W. Ochterski, R. L. Martin, K. Morokuma, V. G. Zakrzewski, G. A. Voth, P. Salvador, J. J. Dannenberg, S. Dapprich, A. D. Daniels, O. Farkas, J. B. Foresman, J. V. Ortiz, J. Cioslowski, D. J. Fox, **2009**.

[12] E. Stendardo, A. Pedone, P. Cimino, M. C. Menziani, O. Crescenzi, V. Barone, *Phys. Chem. Chem. Phys.* **2010**, *12*, 11697-11709.

[13] S. R. Chaudhari, D. Wisser, A. C. Pinon, P. Berruyer, D. Gajan, P. Tordo, O. Ouari, C. Reiter, F. Engelke, C. Copéret, M. Lelli, A. Lesage, L. Emsley, *J. Am. Chem. Soc.* **2017**, *139*, 10609-10612.

[14] D. Wisser, G. Karthikeyan, A. Lund, G. Casano, H. Karoui, M. Yulikov, G. Menzildjian, A. C. Pinon, A. Purea, F. Engelke, S. R. Chaudhari, D. Kubicki, A. J. Rossini, I. B. Moroz, D. Gajan, C. Copéret, G. Jeschke, M. Lelli, L. Emsley, A. Lesage, O. Ouari, *J. Am. Chem. Soc.* **2018**, *140*, 13340-13349.

[15] A. J. Rossini, A. Zagdoun, M. Lelli, D. Gajan, F. Rascon, M. Rosay, W. E. Maas, C. Coperet, A. Lesage, L. Emsley, *Chem. Sci.* **2012**, *3*, 108-115.

[16] I. B. Golovanov, S. M. Zhenodarova, *Russ. J. Gen. Chem.* **2005**, *75*, 1795-1797.

[17] I. Kutsche, G. Gildehaus, D. Schuller, A. Schumpe, *J. Chem. Eng. Data.* **1984**, *29*, 286-287.

[18] a) M. Mardini, R. S. Palani, I. M. Ahmad, S. Mandal, S. K. Jawla, E. Bryerton, R. J. Temkin, S. T. Sigurdsson, R. G. Griffin, *J. Magn. Reson.* **2023**, *353*, 107511; b) L. Delage-Laurin, R. S. Palani, N. Golota, M. Mardini, Y. F. Ouyang, K. O. Tan, T. M. Swager, R. G. Griffin, *J. Am. Chem. Soc.* **2021**, *143*, 20281-20290.

[19] T. V. Can, M. A. Caporini, F. Mentink-Vigier, B. Corzilius, J. J. Walish, M. Rosay, W. E. Maas, M. Baldus, S. Vega, T. M. Swager, R. G. Griffin, *J. Chem. Phys.* **2014**, *141*.

[20] A. Purea, C. Reiter, A. I. Dimitriadis, E. d. Rijk, F. Aussenac, I. Sergeyev, M. Rosay, F. Engelke, *J. Magn. Reson.* **2019**, *302*, 43-49.
